# Anti–PD-L1–IFN-**α**–adjuvanted HBsAg vaccine overcomes HBV immune tolerance through targeting both DCs and macrophages

**DOI:** 10.1172/jci.insight.198097

**Published:** 2025-12-08

**Authors:** Chao-Yang Meng, Yong Liang, Longxin Xu, Hongjia Li, Jingya Guo, Hairong Xu, Fan Wang, Yang-Xin Fu, Hua Peng

**Affiliations:** 1 State Key Laboratory of Biomacromolecules, Institute of Biophysics, Chinese Academy of Sciences, Beijing, China.; 2 Center for Cancer Biology, School of Basic Medical Sciences, Tsinghua University, Beijing, China.; 3 State Key Laboratory of Molecular Oncology, Tsinghua University, Beijing, China.; 4 University of Chinese Academy of Sciences, Beijing, China.; 5 Guangzhou National Laboratory, Guangzhou International Bio-Island, Guangzhou, Guangdong, China.; 6 State Key Laboratory of Respiratory Disease, National Clinical Research Center for Respiratory Disease, Guangzhou Institute of Respiratory Health, the First Affiliated Hospital of Guangzhou Medical University, Guangzhou, Guangdong, China.

**Keywords:** Hepatology, Immunology, Virology, Dendritic cells, Immunotherapy, Macrophages

## Abstract

Recombinant hepatitis B surface antigen (rHBsAg) vaccine with various adjuvants fails to break T and B cell tolerance in hosts with chronic hepatitis B (CHB). This study aims to explore the mechanisms to break immune tolerance that allows the host to respond to rHBsAg, achieving a cure for CHB. We engineered an anti–PD-L1–IFN-α (aPD-L1–IFN-α) heterodimeric fusion protein to allow rHBsAg to rejuvenate T and B cell responses in hepatitis B virus–tolerant (HBV-tolerant) mice. S.c. coimmunization with aPD-L1–IFN-α and rHBsAg significantly enhanced antigen uptake and maturation of both macrophage and dendritic cell (DC) subsets in draining lymph nodes. Macrophages drove early B cell activation, while cDC1s primed CD8^+^ T cells, breaking tolerance and leading to both B cell and cytotoxic T lymphocyte (CTL) differentiation. This strategy elicited not only anti-HBsAg neutralizing antibodies but also HBsAg-specific CD8^+^ T cell responses, achieving a functional cure without systemic toxicity. The efficacy of the aPD-L1–IFN-α adjuvant depended on both PD-L1 *cis*-targeting and IFN-α receptor signaling in antigen-presenting cells. These findings establish aPD-L1–IFN-α as a translatable adjuvant to break the strong tolerance induced by CHB, providing a dual-pathway strategy to induce HBV-specific T and B cell responses.

## Abstract 2

The aPD-L1-IFNα fusion protein adjuvant, when subcutaneously co-administered with HBsAg vaccine, breaks HBV-induced immune tolerance, activates HBsAg-specific immune responses, and offers the potential for achieving a functional cure in chronic hepatitis B.

## Graphical abstract

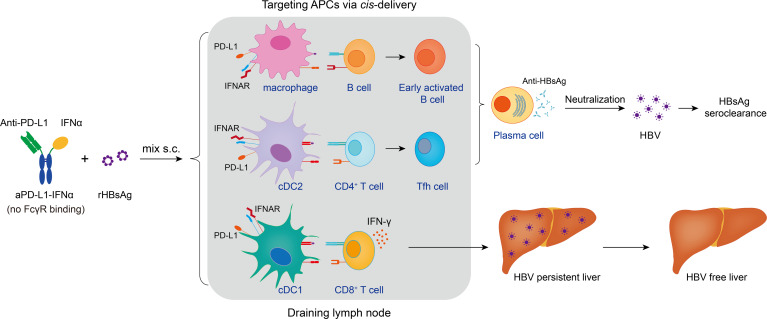

## Introduction

Hepatitis B virus (HBV) is a noncytopathic, hepatotropic DNA virus causing a spectrum of liver diseases, including acute and chronic hepatitis B (CHB), liver fibrosis, cirrhosis, and hepatocellular carcinoma (1, 2). The clearance of HBV largely depends on the adaptive immune responses, including specific CD8^+^ T cell and neutralizing antibody responses, which are often impaired in persistent HBV infections (3, 4). Patients with CHB exhibit immune tolerance to the hepatitis B surface antigen (HBsAg), rendering the recombinant HBsAg (rHBsAg) vaccine ineffective in inducing antiviral responses, thereby limiting its potential as a therapeutic vaccine (5).

Successful HBV clearance necessitates the coordination of innate and adaptive immunity (6, 7). In the lymph nodes (LNs), conventional dendritic cells (cDCs) are classified into resident cDCs (rDCs) and migratory cDCs (mDCs), both further divided into cDC1s and cDC2s (8). DCs, as the most effective antigen-presenting cells (APCs) in vaccine design, have been targeted in previous studies to improve antigen capture and initiate specific immune responses (9, 10). However, merely uptake of antigens by DCs is insufficient to induce effective T cell responses; additional activation signals are required to process and present antigens to T cells effectively (11). Adjuvants play key roles in vaccines, promoting APC activation and maturation, generating costimulatory signals, and enhancing adaptive immunity (12, 13). Dysfunction of DCs during chronic HBV infection might contribute to the immune tolerance of HBV-specific T cells (14). Chronic HBV infection leads to a significant reduction in the allostimulatory capacity of myeloid DCs and a decreased percentage of myeloid DCs expressing costimulatory molecules in patients (15). Moreover, in patients with CHB, the upregulation of programmed death ligand 1 (PD-L1) on DCs is associated with impaired T cell function. Blocking PD-L1 signaling has been shown to enhance the allostimulatory capacity of DCs and promote cytokine production by T cells (16). Thus, blocking PD-L1 signaling and targeting efficient immune adjuvants to APCs in the draining LNs (dLNs) may offer a promising strategy to overcome HBV-induced immune tolerance and enhance rHBsAg vaccine responses.

In the LNs, macrophages are primarily located in the subcapsular sinus and medullary regions, and they are categorized into subcapsular sinus macrophages (SSMs), medullary sinus macrophages (MSMs), and medullary cord macrophages (MCMs) (17, 18). Ferritin nanoparticle-preS1 acts as a scaffold to display multiple copies of the HBV preS1 protein on its outer surface. A previous study showed that macrophages are essential in capturing ferritin nanoparticle-preS1 vaccine, playing a critical role in early B cell activation and immunoglobulin G (IgG) responses against HBV (6). Therefore, it raises the possibility that corporately enhancing DC and macrophage function may improve the immune response to therapeutic vaccines for CHB.

Type I IFNs drive multifaceted immune functions, including the activation and maturation of APCs, enhanced antigen cross-presentation for T cell–mediated immunity, and activation of B cells (19–21). Despite these benefits, short half-life of IFNs and their binding to the ubiquitously expressed IFN-α receptor (IFNAR) often cause severe toxicity at therapeutic doses (22, 23). Our previous study reported that systemic i.v. administration of an anti–PD-L1–IFN-α (aPD-L1–IFN-α) fusion protein (20 μg/dose) could specifically target the liver in HBV carrier mice, thereby providing a potential avenue for overcoming HBV-induced immune tolerance (24). In this study, we observed that PD-L1 expression was significantly higher on APCs than on other cell types in the LNs of HBV carrier mice. Thus, we developed a fusion protein containing a high-affinity aPD-L1 antibody and a low-affinity monomeric IFN-α, engineered to avoid Fcγ receptor (FcγR) binding (aPD-L1–IFN-α), facilitating targeted delivery of IFN-α to APCs in the LN through *cis*-delivery. S.c. local administration of low-dose aPD-L1–IFN-α (2 μg/dose) effectively enhanced antigen capture and presentation functions of APCs, thereby breaking HBV-induced immune tolerance by coordinating the functions of DCs and macrophages in the dLNs.

## Results

### High PD-L1 expression on APCs in LNs facilitates aPD-L1–IFN-α binding.

**Figure 1:**
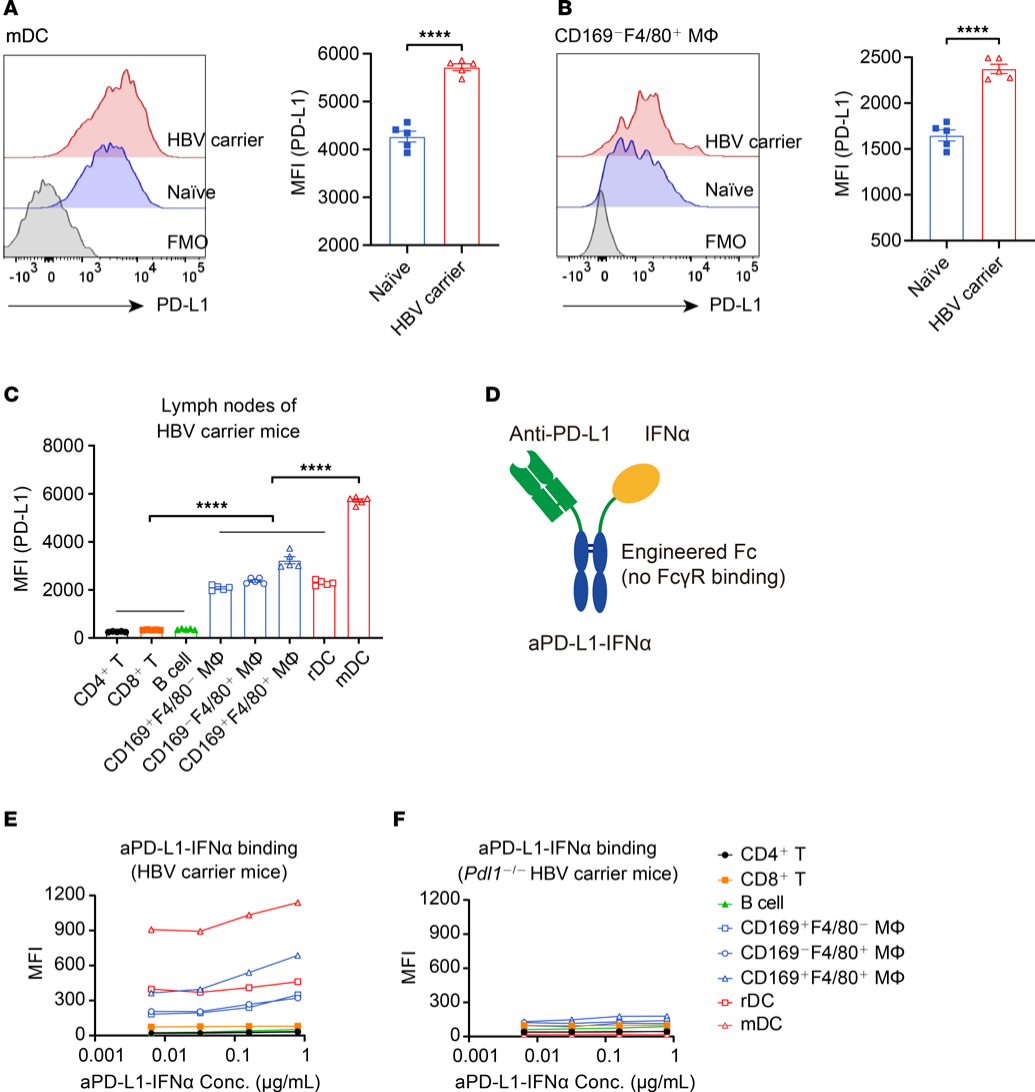
aPD-L1–IFN-α preferentially targets DCs and macrophages. (**A** and **B**) Inguinal LNs of naive or HBV carrier mice (*n* = 5/group) were collected. PD-L1 levels on mDCs (**A**) and CD169^–^F4/80^+^ MΦ (**B**) were assessed by flow cytometry. A representative graph is shown on the left, and the MFI is shown on the right. (**C**) The single cells from inguinal LNs of HBV carrier mice (*n* = 5/group) were collected, and the expression of PD-L1 was analyzed by flow cytometry. PD-L1 expression levels on CD4^+^ T cells (CD3^+^CD4^+^), CD8^+^ T cells (CD3^+^CD8^+^), B cells (B220^+^), macrophages (CD169^+^F4/80^–^, CD169^–^F4/80^+^, CD169^+^F4/80^+^), rDCs (CD11c^hi^MHCII^+^), and mDCs (CD11c^+^MHCII^hi^) are shown. (**D**) Schematic representation of the aPD-L1–IFN-α heterodimeric fusion protein adjuvant with LALA-PG mutants in the fragment crystallizable (Fc) domain. (**E** and **F**) The single cells from inguinal LNs of HBV carrier mice (*n* = 4) (**E**) or *Pdl1*^–/–^ HBV carrier mice (*n* = 4) (**F**) were collected and incubated with aPD-L1–IFN-α (human IgG1 Fc) at the indicated concentrations, followed by incubation with fluorescently labeled antibodies and anti–human IgG Fc (PE-Cy7 label) in vitro. The MFI of PE-Cy7 was detected by flow cytometry. Data are shown as the mean ± SEM and are representative of at least 2 independent experiments. An unpaired 2-tailed Student’s *t* test was applied in **A** and **B**. One-way ANOVA followed by Tukey’s test was applied in **C**. ****P < 0.0001. Conc., concentrations; MΦ, macrophage; MFI, mean fluorescence intensity.

PD-L1 is known to be upregulated on hepatocytes, peripheral myeloid DCs, and intrahepatic DCs in patients with CHB and/or HBV carrier mice (16, 24, 25). In addition to DCs in the liver and peripheral blood, we investigated whether HBV persistence could also induce PD-L1 expression on DCs within the LNs. We examined PD-L1 levels on immune cells in the LNs of HBV carrier mice by flow cytometry. Compared with naive mice, HBV carrier mice had significantly elevated PD-L1 expression on mDCs (CD11c^+^MHCII^hi^) and macrophages (CD11b^+^CD169^–^F4/80^+^) within the LNs (Figure 1, A and B, and gating strategy shown in Supplemental Figure 1, A and B; supplemental material available online with this article; https://doi.org/10.1172/jci.insight.198097DS1). We then profiled PD-L1 expression across various cell types within LNs from HBV carrier mice. PD-L1 expression on DCs (identified as CD11c^hi^MHCII^+^ for rDCs and CD11c^+^MHCII^hi^ for mDCs) and macrophages (characterized as CD11b^+^CD169^+^F4/80^–^ for SSMs, CD11b^+^CD169^–^F4/80^+^ for MCMs, and CD11b^+^CD169^+^F4/80^+^ for MSMs) was higher than that on other cells within the LNs of HBV carrier mice (Figure 1C and Supplemental Figure 1, A and B). While PD-L1 expression on these APCs in HBV carriers could contribute to immune evasion, we wonder whether such high expression might become a target for delivering IFN-α to enhance immune responses.

To take advantage of the high expression of PD-L1 on APCs in LNs, we employed a fusion protein adjuvant that delivers IFN-α to LN APCs by utilizing a heterodimeric format of a high-affinity aPD-L1 antibody fused to a low-affinity monomeric IFN-α (24), and we optimized the heterodimeric fusion protein to eliminate FcγR binding (termed aPD-L1–IFN-α) and to avoid FcγR-mediated effector functions such as antibody-dependent cell-mediated cytotoxicity (ADCC) (Figure 1D). In contrast to the Fc-mutated aPD-L1–IFN-α, the previous fusion protein aPD-L1–IFN-α (WT Fc) resulted in a significantly reduced number of DCs in the dLNs following s.c. administration (Supplemental Figure 1C), suggesting that the Fc-mediated ADCC effect may have led to the depletion of a portion of DCs. High-performance liquid chromatography (HPLC) analysis revealed a single peak, indicating a uniform protein composition of aPD-L1–IFN-α (Supplemental Figure 1D). Additionally, SDS-PAGE confirmed that the purified fusion protein was the expected size and purity (Supplemental Figure 1E).

To address which cells are preferentially targeted by this heterodimeric protein, we assessed the binding affinity of aPD-L1–IFN-α to various immune cells from LNs. Consistent with their top levels of PD-L1 expression, DCs and macrophages demonstrated the highest binding of aPD-L1–IFN-α compared with other immune cells (Figure 1E). Notably, aPD-L1–IFN-α exhibited stronger binding to CD103^+^ mDCs and CD11b^+^ mDCs compared with CD8α^+^ rDCs and CD11b^+^ rDCs (Supplemental Figure 2, A and B). Using *Pdl1*^–/–^ mice, we explored whether this fusion protein targets DCs and macrophages depending on PD-L1 expression. Indeed, aPD-L1–IFN-α did not bind to these immune cells in the absence of PD-L1 (Figure 1F and Supplemental Figure 2, C–G), confirming that the fusion protein primarily targets DCs and macrophages via the PD-L1 molecule, and monomeric IFN-α fails to bind effectively due to its low affinity. This preferential binding to PD-L1^hi^ cells over PD-L1^lo^ IFNAR^+^ cells in LNs underscores the targeted nature of aPD-L1–IFN-α. Taken together, our results demonstrate that PD-L1 is highly expressed on DCs and macrophages in the LNs of HBV carrier mice, facilitating the targeted delivery of aPD-L1–IFN-α to these APCs.

### aPD-L1–IFN-α enhances antigen uptake and presentation by APCs in dLNs.

Initially, we evaluated the effects of several fusion proteins on the activation of DCs in vitro. DC2.4 cells were treated with either aPD-L1, IFN-α–Fc, a combination of aPD-L1 and IFN-α–Fc, or aPD-L1–IFN-α. Notably, aPD-L1–IFN-α treatment led to a significant upregulation of MHC I, CD80, and CD86 on DC2.4 cells, compared with the other treatments (Supplemental Figure 3, A–C). These results suggest that aPD-L1 antibodies profoundly facilitate the binding of IFN-α to DCs, thereby enhancing its bioactivity on PD-L1-positive cells, likely through aPD-L1-mediated *cis*-delivery.

**Figure 2:**
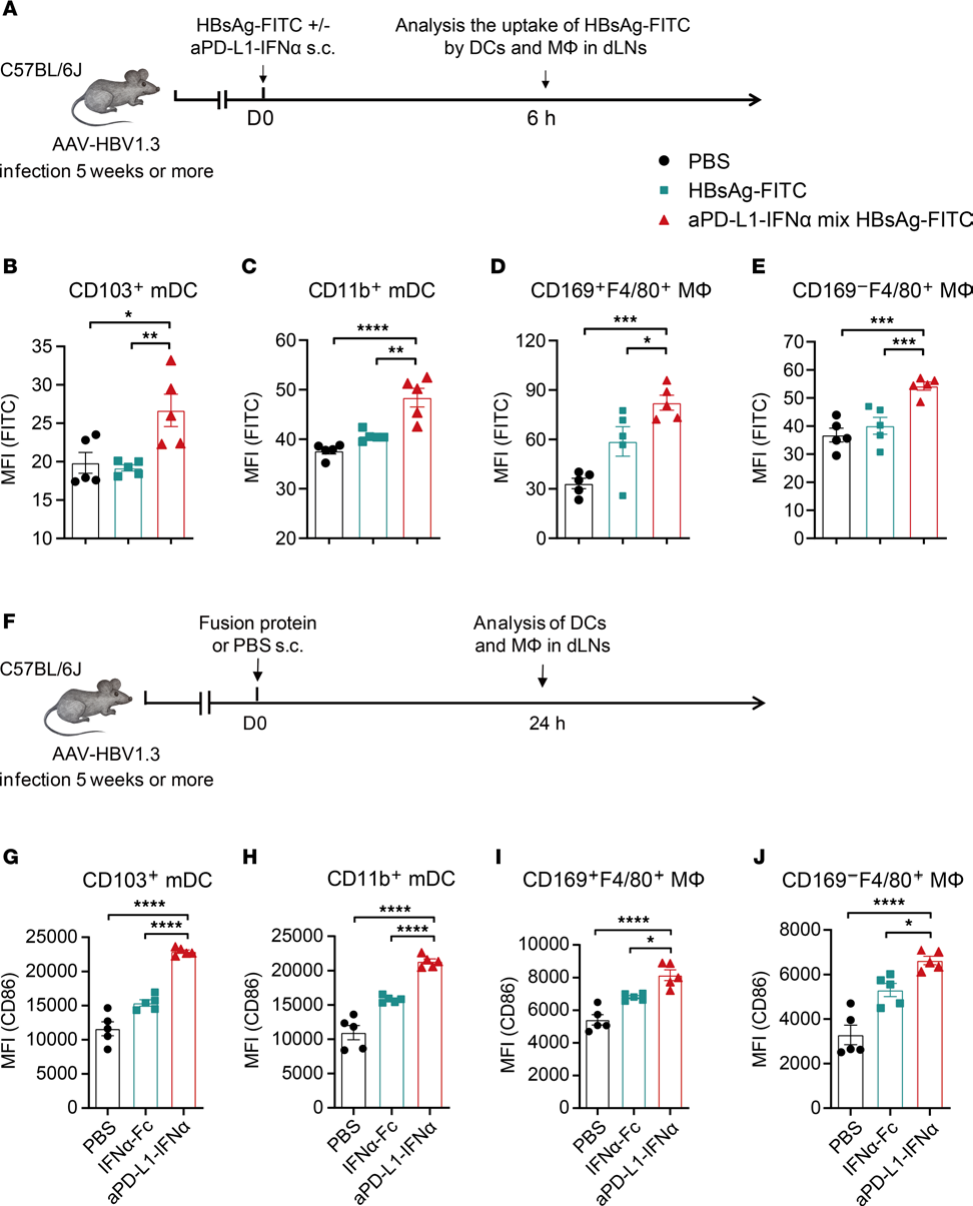
aPD-L1–IFN-α enhances the antigen uptake and maturation of APCs in dLNs. (**A**–**E**) Schematic diagram of s.c. injection of HBsAg-FITC (1 μg/mouse) mix or without aPD-L1–IFN-α (2 μg/mouse) into HBV carrier mice. Six hours after injection, the uptake of HBsAg-FITC by DCs and macrophages in inguinal dLNs was analyzed using flow cytometry (**A**). The uptake of HBsAg-FTIC by CD103^+^ mDCs (**B**), CD11b^+^ mDCs (**C**), CD169^+^F4/80^+^ MΦ (**D**), and CD169^–^F4/80^+^ MΦ (**E**) is shown (*n* = 5/group). (**F**–**J**) Schematic diagram of a single-dose s.c. injection of IFN-α–Fc (0.78 μg/mouse) or aPD-L1–IFN-α (2 μg/mouse) (determined by equimolar of IFN-α subunit) into HBV carrier mice. After 24 hours, the expression of costimulatory molecules on DCs and macrophages in inguinal dLNs was analyzed by flow cytometry (**F**). The expression of CD86 on CD103^+^ mDCs (**G**), CD11b^+^ mDCs (**H**), CD169^+^F4/80^+^ MΦ (**I**), and CD169^–^F4/80^+^ MΦ (**J**) is shown (*n* = 5/group). Data are shown as the mean ± SEM and are representative of at least 2 independent experiments. One-way ANOVA followed by Tukey’s test was applied in **B**–**E** and **G**–**J**. *P < 0.05; **P < 0.01; ***P < 0.001; ****P < 0.0001. FITC, fluorescein isothiocyanate; MΦ, macrophage; MFI, mean fluorescence intensity.

We then investigated the effects of aPD-L1–IFN-α on antigen uptake and activation of APCs in dLNs. HBV carrier mice were administered fluorescently labeled HBsAg (HBsAg-FITC) with or without the fusion protein (Figure 2A). Flow cytometry revealed that aPD-L1–IFN-α significantly enhanced the uptake of HBsAg-FITC by CD103^+^ mDCs, CD11b^+^ mDCs, and macrophages compared with HBsAg-FITC alone (Figure 2, B–E, and Supplemental Figure 4A). However, the uptake by CD8α^+^ rDCs and CD11b^+^ rDCs showed no significant difference between the 2 groups (Supplemental Figure 4, B and C). To assess APC activation, we measured the expression of costimulatory molecules CD80 and CD86 on DCs and macrophages in the dLNs via flow cytometry (Figure 2F). Notably, aPD-L1–IFN-α significantly increased the expression of these costimulatory markers on CD103^+^ mDCs and CD11b^+^ mDCs (Figure 2, G and H, and Supplemental Figure 4, D and E). Importantly, aPD-L1–IFN-α also effectively upregulated the expression of CD80 and CD86 across macrophage subsets, including SSMs, MSMs, and MCMs (Figure 2, I and J, and Supplemental Figure 4, F–I). These data indicate that aPD-L1–IFN-α acts as a targeted adjuvant, potently enhancing the activation of mDCs and macrophages in the dLNs.

### aPD-L1–IFN-α promotes the efficacy of anti-HBV immunity.

To evaluate the efficacy and safety of aPD-L1–IFN-α as an adjuvant in therapeutic vaccine against CHB, we administered aPD-L1–IFN-α mixed with rHBsAg, referred to as the coformulated immunization (coimmunization), via s.c. injection into HBV carrier mice, using aPD-L1, IFN-α–Fc, and aPD-L1 plus IFN-α–Fc mixture as control adjuvants (Supplemental Figure 5A). These fusion proteins alone were unable to induce anti-HBsAg production and reduce HBsAg levels in serum (Supplemental Figure 5, B and C). However, the mixture of aPD-L1–IFN-α with rHBsAg overcame HBV-induced immune tolerance, resulting in a robust anti-HBsAg antibody response and a reduction of serum HBsAg levels. This effect was not observed with the mixture of aPD-L1, IFN-α–Fc, or their combination with rHBsAg (Supplemental Figure 5, D and E), indicating that aPD-L1–IFN-α had superior adjuvant efficacy compared with the nontargeting IFN-α–Fc. Importantly, this coimmunization did not lead to elevated levels of alanine aminotransferase (ALT) or aspartate aminotransferase (AST), nor did it cause weight loss (Supplemental Figure 5, F–I). These results suggest that targeted delivery of IFN-α to APCs in the dLNs via aPD-L1 is the key for breaking HBV-induced immune tolerance, and the aPD-L1–IFN-α–based rHBsAg vaccine is safe for CHB therapy.

**Figure 3:**
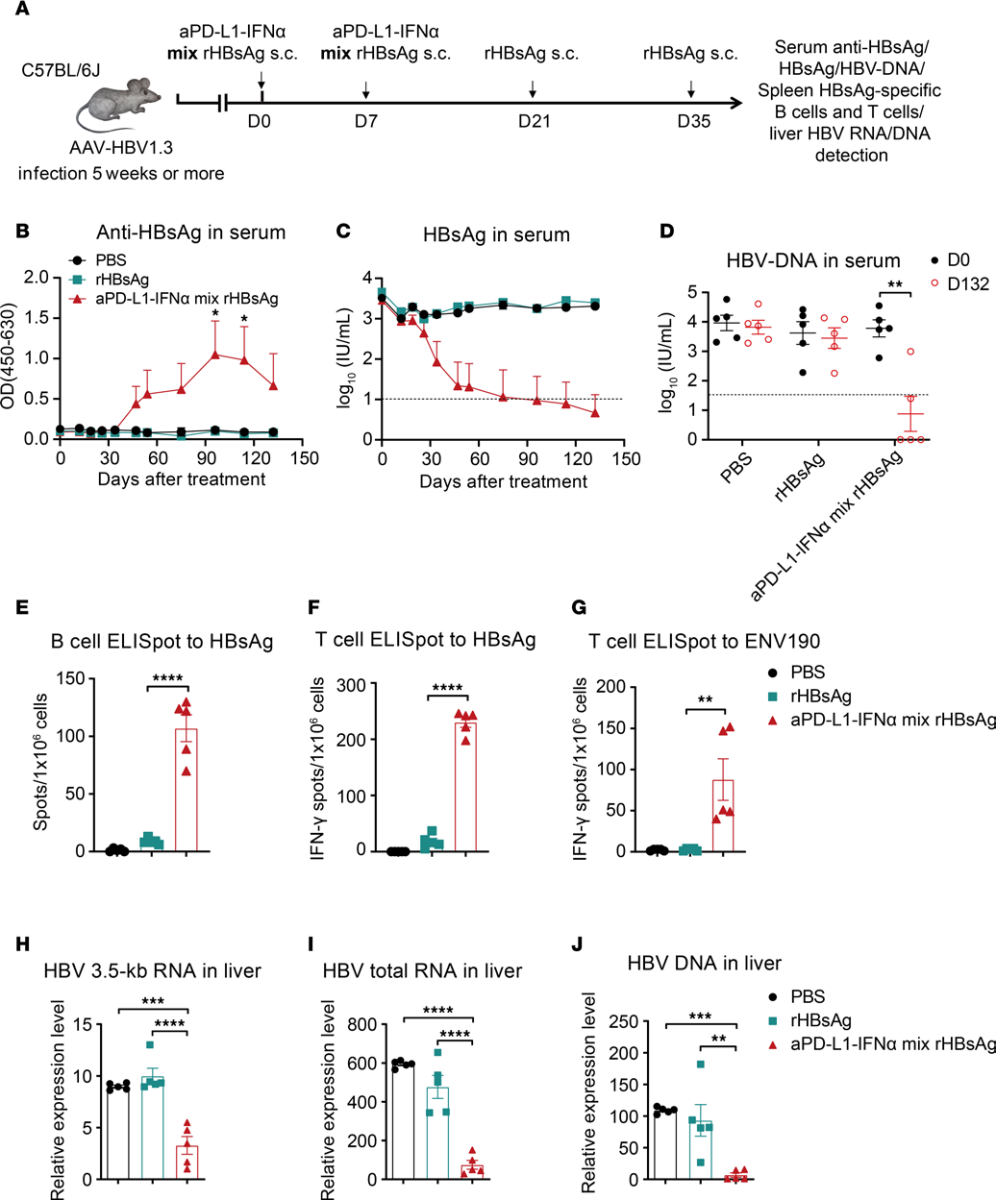
The coimmunization achieves a functional cure in HBV-tolerant mice. (**A**) Time schedule for aPD-L1–IFN-α mixed with rHBsAg as a therapeutic vaccine to treat HBV carrier mice. aPD-L1–IFN-α (2 μg/mouse) was mixed with rHBsAg (1 μg/mouse) (denoted as aPD-L1–IFN-α mix rHBsAg, coimmunization) and injected s.c. into HBV carrier mice. A second immunization with the same mixture was given 7 days after the initial immunization, followed by booster immunizations with rHBsAg alone at 3 and 5 weeks after initial immunization. The control group received rHBsAg (1 μg/mouse) s.c. injection into HBV carrier mice on days 0, 7, 21, and 35. (**B** and **C**) Serum levels of ayw subtype–specific anti-HBsAg IgG (**B**) and HBsAg (**C**) (*n* = 5/group) were examined by ELISA. (**D**) Serum levels of HBV-DNA (*n* = 5/group) were determined by qPCR. The detection limits in **C** and **D** are indicated by dashed lines. (**E**–**G**) A total of 1 × 10^6^ splenocytes from each mouse (*n* = 5/group) were collected on day 132. Specific B cell (**E**) and T cell (**F**) responses to HBsAg (subtype ayw) were tested by B cell and T cell ELISpot assays, respectively. ENV190-specific CD8^+^ T cell responses were tested by a T cell ELISpot assay (**G**). (**H**–**J**) Levels of HBV intermediate products, including HBV 3.5-kb RNA (**H**), HBV total RNA (**I**), and HBV DNA (**J**) in the liver, were measured with real-time PCR at the end of the experiment (*n* = 5/group). Data are shown as the mean + SEM (**B** and **C**) or mean ± SEM (**D**–**J**) and are representative of at least 2 independent experiments. An unpaired 2-tailed Student’s *t* test was applied in **D**–**G**. One-way ANOVA followed by Tukey’s test was applied in **B** and **H**–**J**. **P < 0.01; ***P < 0.001; ****P < 0.0001. OD, optical density; s.c., s.c.

We further assessed the therapeutic efficacy and immune responses induced by the coimmunization in HBV-tolerant mice (Figure 3A). Compared with other groups, mice receiving the coimmunization exhibited a significantly stronger anti-HBsAg antibody response, along with a continuous decrease in serum HBsAg levels and undetectable HBV-DNA in 3 of 5 mice (Figure 3, B–D), suggesting potential for a functional cure (defined as serological negativity for HBsAg and HBV-DNA). Additionally, the coimmunization elicited robust HBsAg-specific B cell responses (Figure 3E). Notably, splenocytes from HBV-tolerant mice treated with the coimmunization showed significant numbers of IFN-γ spots upon ex vivo stimulation with the HBsAg antigen (subtype ayw) (Figure 3F). Furthermore, there was a strong induction of HBsAg-specific IFN-γ producing CD8^+^ T cells following ex vivo stimulation with ENV190, an HBsAg-specific CD8 peptide (Figure 3G). Crucially, real-time PCR analysis showed a significant reduction in HBV-RNA and HBV DNA loads in the liver of the coimmunization group (Figure 3, H–J). These results demonstrate that aPD-L1–IFN-α effectively enhances HBsAg-specific immune responses and reduces HBV levels when coadministered with rHBsAg, highlighting its potential as an effective adjuvant for CHB therapy.

### aPD-L1–IFN-α breaks immune tolerance to rHBsAg via DC and macrophage coordination.

**Figure 4:**
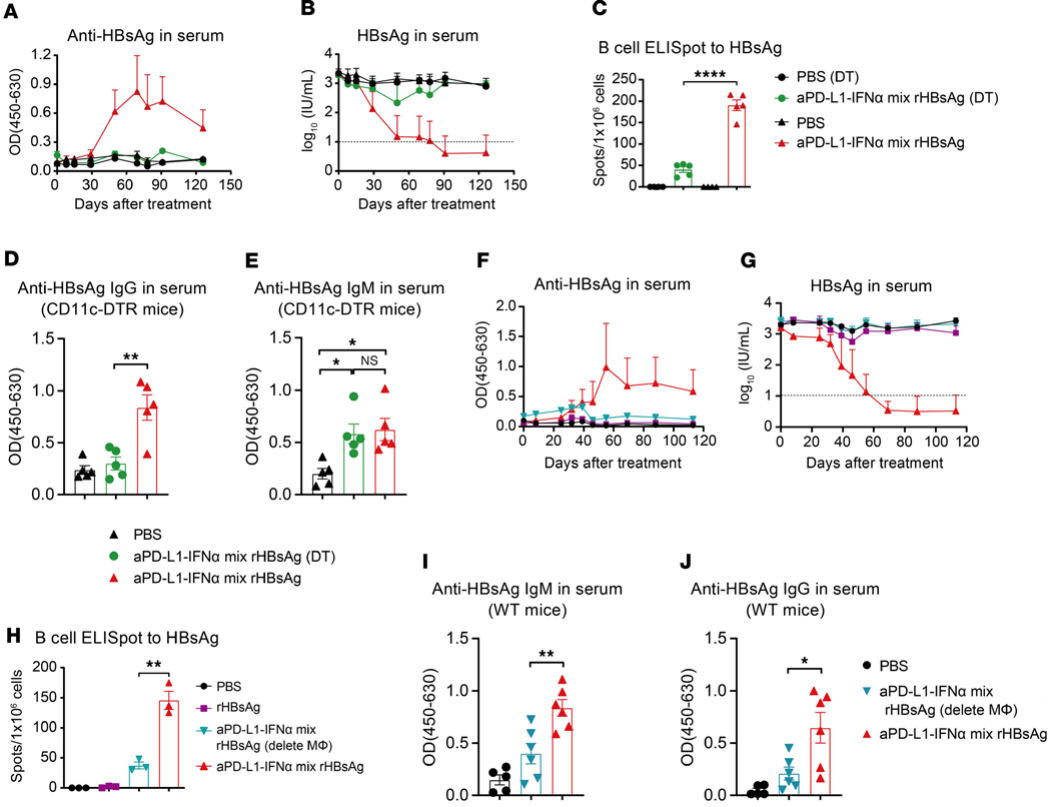
aPD-L1–IFN-α breaks immune tolerance to rHBsAg vaccine through coordinating DCs and macrophages. (**A**–**C**) HBV carrier CD11c-DTR bone marrow chimeric mice were generated and treated as described in Figure 3A. For DC depletion, DT (4 ng/g body weight) was administered i.p. every 2 days starting 24 hours before immunization. Serum levels of ayw subtype–specific anti-HBsAg IgG (**A**) and HBsAg (**B**) (*n* = 4–5/group) were determined by ELISA. The detection limit in **B** is indicated by a dashed line. A total of 1 × 10^6^ splenocytes from each mouse (*n* = 4–5/group) were collected on day 126. Specific B cell responses to HBsAg (subtype ayw) were tested by a B cell ELISpot assay (**C**). (**D** and **E**) CD11c-DTR bone marrow chimeric mice (*n* = 5/group) were immunized s.c. with the mixture of aPD-L1–IFN-α (2 μg/mouse) and rHBsAg (1 μg/mouse) on days 0 and 14. For DC depletion, DT (4 ng/g body weight) was administered i.p. every 2 days starting 24 hours before immunization. HBsAg-specific IgG (**D**) and IgM (**E**) responses were measured on day 21 and day 10 after the first immunization, respectively. (**F**–**H**) HBV carrier mice were treated as described in Figure 3A. For macrophage depletion in inguinal LNs, CLL (40 μL/mouse) was injected s.c. every 3 days, starting 7–10 days before the first immunization. Serum levels of ayw subtype–specific anti-HBsAg IgG (**F**) and HBsAg (**G**) (*n* = 3/group) were determined using ELISA. The detection limit in **G** is indicated by a dashed line. A total of 1 × 10^6^ splenocytes from each mouse (*n* = 3/group) were collected on day 113. Specific B cell responses to HBsAg (subtype ayw) were assessed by a B cell ELISpot assay (**H**). (**I** and **J**) WT C57BL/6J mice (*n* = 5–6/group) were immunized s.c. with the mixture of aPD-L1–IFN-α (2 μg/mouse) and rHBsAg (1 μg/mouse) on days 0 and 14. For macrophage depletion in inguinal LNs, CLL (40 μL/mouse) was injected s.c. every 3 days, starting 7–10 days before the first immunization. HBsAg-specific IgM (**I**) and IgG (**J**) responses were measured on day 10 and day 21 after the first immunization, respectively. Data are shown as the mean + SEM (**A**, **B**, **F**, and **G**) or mean ± SEM (**C**–**E** and **H**–**J**) and are representative of at least 2 independent experiments. An unpaired 2-tailed Student’s *t* test was applied in **C**, **D**, and **H**–**J**. One-way ANOVA followed by Tukey’s test was applied in **E**. *P < 0.05; **P < 0.01; ****P < 0.0001. MΦ, macrophage; OD, optical density.

We then evaluated the role of DCs in overcoming HBV-induced immune tolerance using HBV carrier CD11c-DTR (diphtheria toxin [DT] receptor) bone marrow chimeric mice. DT significantly reduced the DC level in vivo (Supplemental Figure 6, A and B). Following DT treatment, the coimmunization failed to elicit anti-HBsAg IgG antibodies and to reduce serum HBsAg levels effectively, and HBsAg-specific B cell responses were also markedly diminished (Figure 4, A–C), highlighting the critical role of DCs in antibody responses. Similar results were found in CD11c-DTR bone marrow chimeric mice (non-HBV carrier), where DC depletion resulted in a lower titer of IgG response (Figure 4D). In contrast, levels of IgM antibodies, which are primarily produced by early-activated B cells in a T cell–independent manner (26), remained unaffected (Figure 4E), indicating that DCs are not essential for early B cell activation during the coimmunization. Next, we evaluated the effect of aPD-L1–IFN-α on the function of CD103^+^ mDCs (cDC1s), which are crucial for antigen cross-presentation (27). Encouragingly, aPD-L1–IFN-α significantly enhanced the antigen cross-presentation capability of CD103^+^ mDCs (Supplemental Figure 7, A–C). In *Batf3*^–/–^ HBV carrier mice, which lack CD103^+^ mDCs, the coimmunization resulted in reduced HBsAg-specific IFN-γ producing CD8^+^ T cells and had a limited effect on HBV-RNA and HBV DNA levels in the liver (Supplemental Figure 7, D–I). In addition, the coimmunization failed to clear HBsAg in the serum (Supplemental Figure 7J). These findings underscore the importance of CD103^+^ mDCs in mediating CD8^+^ T cell responses, which are pivotal for HBV elimination.

We further investigated the role of macrophages during the coimmunization. HBV carrier mice were treated with clodronate liposomes (CLL), which significantly reduced macrophage levels in LNs (Supplemental Figure 8, A–D). CLL treatment substantially impaired the coimmunization-induced anti-HBsAg IgG antibodies, serum HBsAg reduction, and HBsAg-specific B cell responses (Figure 4, F–H). Interestingly, CLL treatment also significantly reduced the anti-HBsAg IgM levels on day 10 after the first immunization, further decreasing anti-HBsAg IgG levels on day 21 in CLL-treated mice (Figure 4, I and J). These results indicate that macrophages are vital for early B cell activation and subsequent IgG responses. Collectively, these data suggest that both DCs and macrophages are essential for overcoming HBV-induced immune tolerance by coimmunization, coordinating to induce necessary humoral immune responses for neutralizing serum HBV.

We finally examined the effect of aPD-L1–IFN-α on germinal center (GC) formation, which is pivotal for potent antibody responses, and the affinity maturation of GC B cells depends on T follicular helper (Tfh) cells (28). Impressively, the coimmunization significantly increased the percentages and numbers of Tfh cells (CD4^+^CXCR5^+^PD1^+^) (Supplemental Figure 9, A, C, and D) and GC B cells (B220^+^FAS^+^GL7^+^) (Supplemental Figure 9, B, E, and F) in the dLNs of HBV carrier mice compared with rHBsAg alone. Thus, aPD-L1–IFN-α can serve as an effective adjuvant-delivery platform, enhancing Tfh and GC B cell responses in the dLNs when coformulated with the rHBsAg vaccine.

### aPD-L1–IFN-α specifically activates PD-L1-expressing APCs via cis-binding.

**Figure 5:**
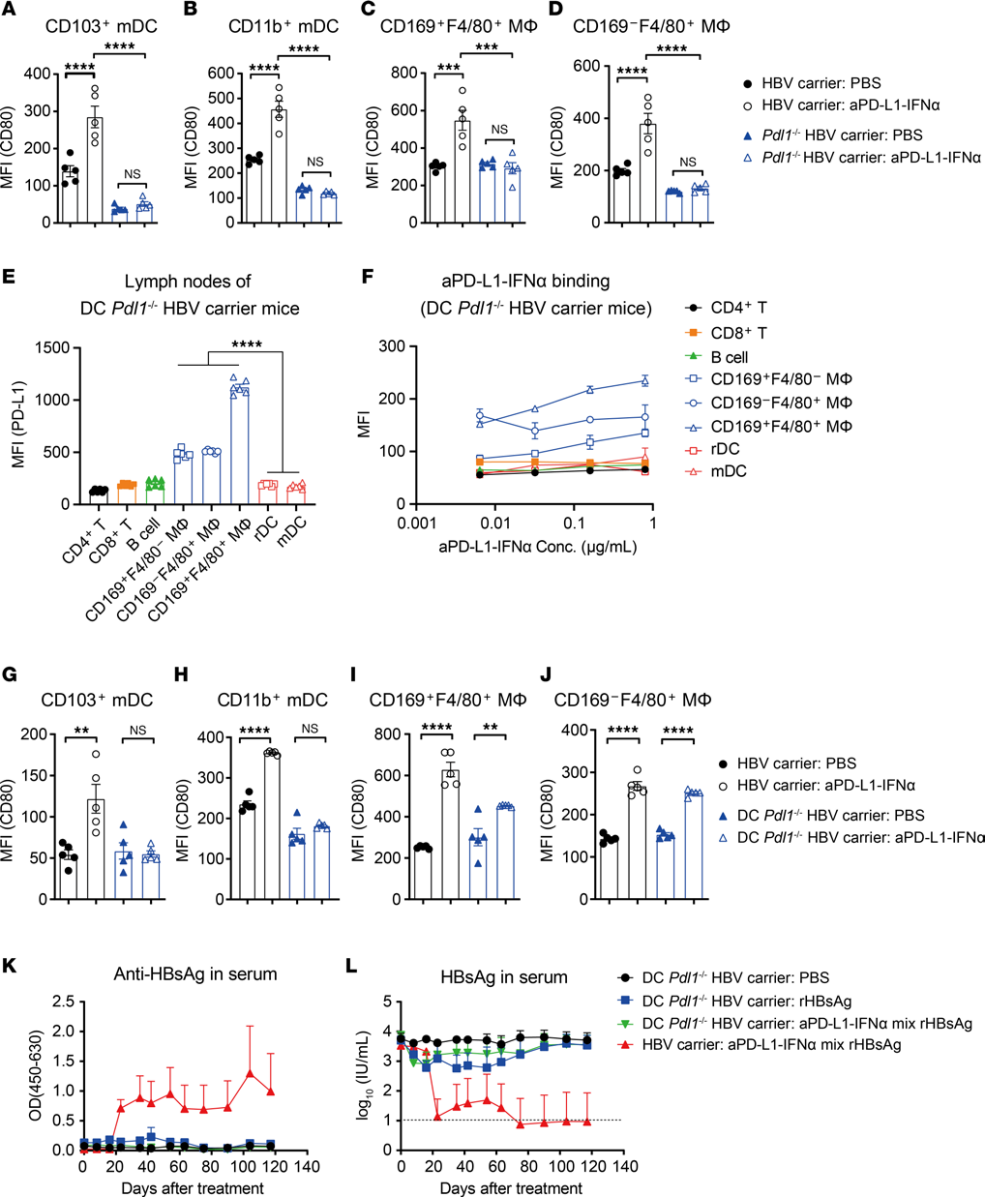
aPD-L1–IFN-α overcomes immune tolerance by aPD-L1-mediated *cis*-targeting. (**A**–**D**) HBV carrier mice or *Pdl1*^–/–^ HBV carrier mice (*n* = 5/group) were injected s.c. with a single dose of aPD-L1–IFN-α (2 μg/mouse) or PBS. After 24 hours, the expression of CD80 on DCs and macrophages in inguinal dLNs was analyzed by flow cytometry. The expression of CD80 on CD103^+^ mDCs (**A**), CD11b^+^ mDCs (**B**), CD169^+^F4/80^+^ MΦ (**C**), and CD169^–^F4/80^+^ MΦ (**D**) is shown. (**E** and **F**) Inguinal LNs of DC *Pdl1*^–/–^ HBV carrier mice (*n* = 6) were collected and analyzed by flow cytometry. PD-L1 expression levels on CD4^+^ T cells, CD8^+^ T cells, B cells, macrophages, rDCs, and mDCs are shown (**E**). Single cells from inguinal LNs of DC *Pdl1*^–/–^ HBV carrier mice (*n* = 3) were collected and incubated with aPD-L1–IFN-α (human IgG1 Fc) at the indicated concentrations, followed by incubation with fluorescently labeled antibodies and anti–human IgG Fc (PE-Cy7 label) in vitro. The MFI of PE-Cy7 was detected by flow cytometry (**F**). (**G**–**J**) HBV carrier mice or DC *Pdl1*^–/–^ HBV carrier mice (*n* = 5/group) were injected s.c. with a single dose of aPD-L1–IFN-α (2 μg/mouse) or PBS. After 24 hours, the expression of CD80 on DCs and macrophages in inguinal dLNs was analyzed by flow cytometry. The expression of CD80 on CD103^+^ mDCs (**G**), CD11b^+^ mDCs (**H**), CD169^+^F4/80^+^ MΦ (**I**) and CD169^–^F4/80^+^ MΦ (**J**) is shown. (**K** and **L**) HBV carrier mice or DC *Pdl1*^–/–^ HBV carrier mice (*n* = 3/group) were treated as described in Figure 3A. Serum levels of ayw subtype–specific anti-HBsAg IgG (**K**) and HBsAg (**L**) were determined by ELISA. The detection limit in **L** is indicated by a dashed line. Data are shown as the mean ± SEM (**A**–**J**) or mean + SEM (**K** and **L**) and are representative of at least 2 independent experiments. One-way ANOVA followed by Tukey’s test was applied in **A**–**E** and **G**–**J**) **P < 0.01; ***P < 0.001; ****P < 0.0001. Conc., concentrations; MΦ, macrophage; MFI, mean fluorescence intensity; OD, optical density.

The *cis*-delivery of low-affinity cytokines to T cells has been shown to be highly potent in activating T cells (29–31). To investigate whether aPD-L1 can directly target APCs via *cis*-binding, we compared the activation levels of WT and PD-L1–KO APCs treated with aPD-L1–IFN-α. In HBV carrier mice, aPD-L1–IFN-α treatment significantly increased the costimulatory molecule CD80 expression on WT APCs, indicating enhanced activation. Conversely, in *Pdl1*^–/–^ HBV carrier mice, aPD-L1–IFN-α failed to upregulate CD80 on PD-L1–KO APCs (Figure 5, A–D). These data suggest that aPD-L1–mediated binding successfully restored the bioactivity of IFN-α specifically on PD-L1^hi^ APCs. These findings confirm that aPD-L1–IFN-α preferentially activates PD-L1^hi^ APCs through a *cis*-binding mechanism.

We further investigated the specific role of PD-L1 on DCs using DC-conditional PD-L1–KO (DC *Pdl1*^–/–^) HBV carrier mice. In these mice, aPD-L1–IFN-α did not bind to DCs but could bind to macrophages (Figure 5, E and F). Consequently, aPD-L1–IFN-α failed to activate DCs but was able to activate macrophages in the dLNs of DC *Pdl1*^–/–^ HBV carrier mice, as shown by CD80 expression levels (Figure 5, G–J). This suggests that PD-L1 on DCs is crucial for the *cis*-targeting by aPD-L1–IFN-α. To delineate the importance of PD-L1 on DCs in breaking HBV-induced immune tolerance, DC *Pdl1*^–/–^ HBV carrier mice were treated with a mixture of aPD-L1–IFN-α and rHBsAg. The coimmunization did not achieve a functional cure in these mice (Figure 5, K and L), underscoring the essential role of PD-L1 on DCs for the efficacy of aPD-L1–IFN-α targeted adjuvant function.

### aPD-L1–IFN-α’s immunoadjuvant function depends on IFNAR signaling in DCs and macrophages.

The role of IFN-α in the aPD-L1–IFN-α was investigated using *Ifnar1*^–/–^ HBV carrier mice. Remarkably, in these mice, aPD-L1–IFN-α was unable to enhance CD80 expression on mDCs and macrophages in the dLNs (Supplemental Figure 10, A–D). Additionally, the coimmunization was ineffective in generating anti-HBsAg antibodies and reducing serum HBsAg levels (Supplemental Figure 10, E and F), with a significant decline in HBsAg-specific T cell responses in *Ifnar1*^–/–^ HBV carrier mice (Supplemental Figure 10G). These data demonstrate that aPD-L1–IFN-α modulates immune responses through the IFNAR signaling pathway.

**Figure 6:**
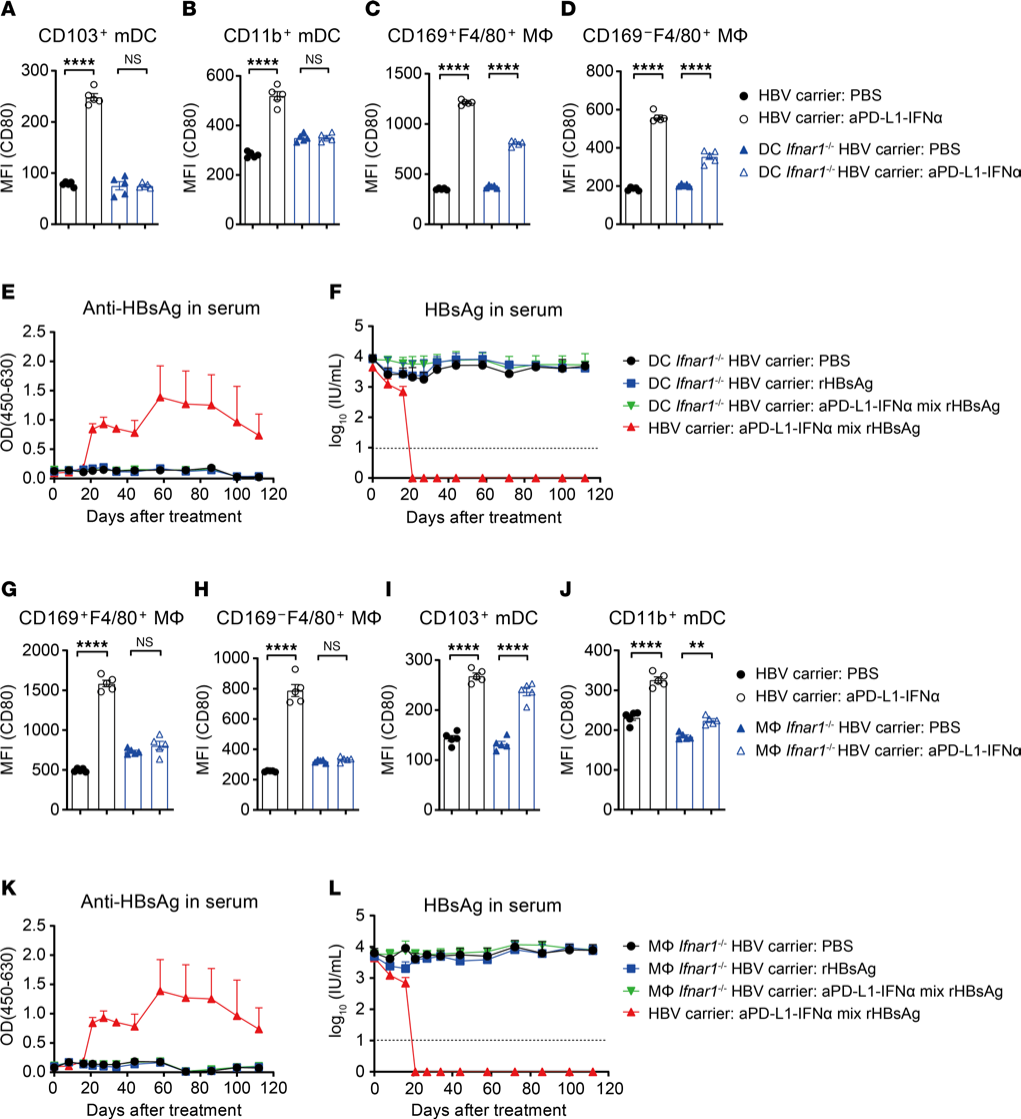
aPD-L1–IFN-α exerts immunoadjuvant activity through the IFNAR signaling pathway in DCs and macrophages. (**A**–**D**) HBV carrier mice or DC *Ifnar1*^–/–^ HBV carrier mice (*n* = 5/group) were injected s.c. with a single dose of aPD-L1–IFN-α (2 μg/mouse) or PBS. After 24 hours, the expression of CD80 on DCs and macrophages in inguinal dLNs was analyzed by flow cytometry. The expression of CD80 on CD103^+^ mDCs (**A**), CD11b^+^ mDCs (**B**), CD169^+^F4/80^+^ MΦ (**C**), and CD169^–^F4/80^+^ MΦ (**D**) is shown. (**E** and **F**) HBV carrier mice or DC *Ifnar1*^–/–^ HBV carrier mice (*n* = 3/group) were treated as described in Figure 3A. Serum levels of ayw subtype–specific anti-HBsAg IgG (**E**) and HBsAg (**F**) were determined by ELISA. The detection limit in **F** is indicated by a dashed line. (**G**–**J**) HBV carrier mice or macrophage *Ifnar1*^–/–^ HBV carrier mice (*n* = 5/group) were injected s.c. with a single dose of aPD-L1–IFN-α (2 μg/mouse) or PBS. After 24 hours, the expression of CD80 on DCs and macrophages in inguinal dLNs was analyzed by flow cytometry. The expression of CD80 on CD169^+^F4/80^+^ MΦ (**G**), CD169^–^F4/80^+^ MΦ (**H**), CD103^+^ mDCs (**I**), and CD11b^+^ mDCs (**J**) is shown. (**K** and **L**) HBV carrier mice or macrophage *Ifnar1*^–/–^ HBV carrier mice (*n* = 3/group) were treated as described in Figure 3A. Serum levels of ayw subtype–specific anti-HBsAg IgG (**K**) and HBsAg (**L**) were determined by ELISA. The detection limit in **L** is indicated by a dashed line. **E**, **F**, **K**, and **L** represent the same batch of experiments. Data are shown as the mean ± SEM (**A**–**D** and **G**–**J**) or mean + SEM (**E**, **F**, **K**, and **L**) and are representative of at least 2 independent experiments. One-way ANOVA followed by Tukey’s test was applied in **A**–**D** and **G**–**J**. **P < 0.01; ****P < 0.0001. MΦ, macrophage; MFI, mean fluorescence intensity; NS, not significant; OD, optical density.

To further determine the role of IFNAR1 on DCs, we generated DC-conditional IFNAR1-KO (DC *Ifnar1*^–/–^) mice by crossing *Ifnar1*^fl/fl^ mice with *Zbtb46*^cre^ mice. In these mice, aPD-L1–IFN-α could not upregulate costimulatory molecules on DCs but did so on macrophages (Figure 6, A–D). Treating DC *Ifnar1*^–/–^ HBV carrier mice with aPD-L1–IFN-α and rHBsAg did not achieve a functional cure (Figure 6, E and F). Similarly, to explore the function of IFNAR1 on macrophages, we generated macrophage-conditional IFNAR1-KO (macrophage *Ifnar1*^–/–^) mice by crossing *Ifnar1*^fl/fl^ mice with *Lyz2*^cre^ mice. In these mice, aPD-L1–IFN-α failed to upregulate costimulatory molecules on macrophages but succeeded on DCs (Figure 6, G–J). The coimmunization in macrophage *Ifnar1*^–/–^ HBV carrier mice also did not achieve a functional cure (Figure 6, K and L). Thus, these results collectively indicate that aPD-L1–IFN-α exerts its immune activation effects through the IFNAR signaling pathway in both DCs and macrophages.

## Discussion

Current CHB therapies rarely achieve functional cure due to persistent immune tolerance. Several studies have highlighted the potential role of cytokines as adjuvants in therapeutic HBV vaccines (32, 33). We propose that *cis*-delivering IFN-α to LN APCs could overcome HBV-induced immune tolerance. However, IFN-driven expression of the immunosuppressive molecule PD-L1 limits IFN-induced immunity. To address this, we engineered the aPD-L1–IFN-α that specifically delivers IFN-α to PD-L1^hi^ APCs, including DCs and macrophages, in the dLNs. This approach effectively restores the bioactivity of IFN-α on APCs through a *cis*-binding interaction facilitated by aPD-L1 antibodies. Targeting IFN-α to APCs by aPD-L1 antibody establishes a feedforward loop, enhancing antigen uptake and presentation by APCs, thereby stimulating HBV-specific T and B cell responses. This strategy effectively breaks HBV-induced immune tolerance, paving the way for achieving a functional cure.

In patients with CHB, HBsAg-specific B cells are often dysfunctional, and anti-HBsAg antibodies are typically undetectable (4, 5). Effective antibody responses begin with antigen interaction with B cells, which primarily reside in the lymphoid follicles of secondary lymphoid tissues (34). Large amounts of antigen are captured and processed by macrophages in the LN medulla, while smaller amounts are captured by SSMs (18, 34). Macrophages play a crucial role in presenting antigens to B cells, thereby facilitating their early activation and memory B cell recall response (6, 35, 36). Our findings in HBV-tolerant mice reveal that aPD-L1–IFN-α significantly enhances rHBsAg uptake by medullary and subcapsular macrophages in the dLNs while upregulating costimulatory molecule expression, potentially presenting antigens to B cells for early activation. The production of IgM antibodies, independent of Tfh cell assistance, directly reflects early B cell activation (26). Depletion of macrophages resulted in significantly reduced anti-HBsAg IgM and IgG levels, underscoring the vital role of macrophages in early B cell activation and subsequent IgG responses. Further investigation is needed to identify the specific roles of each macrophage subset in the LNs during coimmunization. Nonetheless, enhancing macrophage-mediated capture and presentation of rHBsAg to activate B cells presents an effective strategy for improving therapeutic vaccines against CHB.

Early-activated B cells require Tfh cells for their further differentiation. cDC2s are pivotal in activating CD4^+^ T cells to differentiate into Tfh cells, which migrate into B cell follicles to promote the differentiation of early-activated B cells into plasma cells and initiate the GC reaction, leading to the production of high affinity neutralizing antibodies (8, 28). Deficiencies in Tfh cells and their secreted cytokines impair normal B cell responses, leading to humoral immune deficiencies and chronic HBV infection (37, 38). Encouragingly, our study demonstrated that aPD-L1–IFN-α can target and activate CD11b^+^ mDCs (cDC2s), thereby improving rHBsAg uptake and costimulatory signaling to promote Tfh cell and GC B cell generation in the dLNs of HBV-tolerant mice.

The cDC1s are known for cross-presenting antigens to activate CD8^+^ T cells (8), which play a crucial role in HBV clearance through the production of antiviral cytokines and elimination of infected hepatocytes (39, 40). In our study, we found that aPD-L1–IFN-α also targets and activates CD103^+^ mDCs (cDC1s), thereby enhancing rHBsAg uptake, cross-presentation, and costimulatory signaling to induce HBsAg-specific CD8^+^ T cells, which in turn reduces HBV levels in the liver.

This study introduces a therapeutic strategy that is conceptually distinct from our prior work (24). The prior study employed i.v. administration of a high-dose (20 μg/mouse) aPD-L1–IFN-α fusion protein, targeting the liver to directly reduce viral load and activate intrahepatic DCs, thereby establishing an environment conducive to breaking liver-induced systemic immune tolerance in HBV carrier mice. Subsequent s.c. immunization with an HBsAg/CpG vaccine resulted in a functional cure. The primary focus of the previous research was to elucidate the fusion protein’s mechanism of action in the liver. In contrast, the current study utilized a low-dose (2 μg/mouse) aPD-L1–IFN-α fusion protein, which was mixed directly with an aluminum-adjuvanted rHBsAg vaccine for s.c. immunization. This approach targets DCs and macrophages within the dLNs to enhance their functions, promote the rHBsAg vaccine-induced immune response, generate a robust HBV-specific immune response, and ultimately achieve a functional cure for CHB. The primary focus of this study was to elucidate the fusion protein’s mechanism of action in the dLNs. Furthermore, the fusion protein used in this study was optimized to avoid FcγR-mediated effector functions. This coimmunization strategy offers greater operational simplicity, a lower required dose, and the use of an aluminum adjuvant, collectively providing enhanced potential for clinical translation.

The aPD-L1–IFN-α fusion protein, by enabling *cis*-targeting and localized delivery of IFN-α to PD-L1^hi^ APCs in the dLNs, synergizes with the rHBsAg vaccine to achieve a functional cure for chronic HBV infection, while demonstrating a favorable preclinical safety profile. For clinical translation, a localized, low-frequency s.c. administration regimen is recommended to restrict systemic exposure and enhance safety. Pharmacodynamic monitoring of DC and macrophage activation in patient LNs or peripheral blood may serve as predictive biomarkers to guide treatment. Given the immune-activating mechanism, clinical advancement necessitates the establishment of a risk prediction model based on autoimmunity-related biomarkers and medical history for patient stratification (41, 42). Future clinical trials should incorporate risk stratification and long-term follow-up to ensure a favorable benefit-to-risk ratio in human patients.

The AAV-HBV model has served as a valuable platform for investigating immune activation and evaluating therapeutic vaccines (43, 44), as it efficiently establishes a state of high antigenemia and immune tolerance. The findings that aPD-L1–IFN-α can break this tolerance and enhance vaccine-induced immunity are robust and mechanistically insightful within this system. As a noninfectious model, it cannot assess the efficacy of therapeutic strategies in controlling viral transmission or reinfection (45) and, thus, is unable to fully replicate a natural viral infection.

In conclusion, by analyzing responses in various KO and conditional KO mouse strains, this study elucidates how the aPD-L1–IFN-α adjuvant specificity for PD-L1^hi^ APCs in the dLNs ensures *cis*-targeted immune stimulation to activate the innate immune system and modulate adaptive immunity effectively. Notably, the adjuvant’s efficacy hinges on the coordinated functions of DCs and macrophages; macrophages drive early B cell activation (IgM production), while mDCs prime Tfh and CD8^+^ T cells for sustained immunity. By harnessing PD-L1 targeting and localized IFN-α activity, the aPD-L1–IFN-α adjuvant helps break HBV-induced immune tolerance, restore HBsAg-specific immunity, and achieve a functional cure in a preclinical model. These findings not only enhance our understanding of this targeted adjuvant but also inform the design of future vaccine adjuvants, offering a promising strategy for improving therapeutic vaccines against CHB.

## Methods

Additional methods are provided in the Supplemental Material.

### Sex as a biological variable.

Our study examined male and female animals, and comparable results were observed across both sexes. However, we utilized male mice in most of the experiments because male animals exhibited a more stable HBV immune tolerance phenotype.

### Study design.

This study aims to investigate whether activating APCs in the dLNs can break HBV-induced immune tolerance. We engineered an aPD-L1–IFN-α heterodimeric fusion protein as an adjuvant, which was mixed with rHBsAg and s.c. coadministered to HBV-tolerant mice. The aPD-L1–IFN-α *cis-*targeted low-affinity IFN-α to dLN APCs through high-affinity aPD-L1 antibody, activating APCs to promote rHBsAg immunity. The cellular and molecular mechanisms of the aPD-L1–IFN-α fusion protein’s adjuvant activity were investigated through cell depletion experiments and gene KO mouse models.

### Mice and an HBV carrier mouse model.

WT C57BL/6J male mice were purchased from SiPeiFu (SPF) (Beijing) Biotechnology (Beijing, China). *Zbtb46*^Cre^ mice, *Batf3*^–/–^ mice and CD11c-DTR mice were purchased from The Jackson Laboratory. *Ifnar1*^fl/fl^ mice were generated in Shanghai Model Organisms. DC conditional PD-L1–KO (DC *Pdl1*^–/–^) mice, *Pdl1*^–/–^ mice and *Ifnar1*^–/–^ mice were provided by H. Tang (Tsinghua University, Beijing, China). *Lyz2*^Cre^ mice were provided by X. Hu (Tsinghua University, Beijing, China). OT-I TCR (T cell antigen receptor) transgenic male mice were provided by M. Zhu (Institute of Microbiology, Chinese Academy of Sciences, Beijing, China). All mice used in the experiments were 6–8 weeks old and on the C57BL/6 background. For the use of KO and conditional KO mice, age- and sex-matched mice were used for each experiment. Mice were housed under specific pathogen–free conditions in an ABSL-2 animal facility at the Institute of Biophysics, Chinese Academy of Sciences.

The adeno-associated virus (AAV)-HBV1.3 virus was purchased from PackGene Biotech (Guangzhou, China). This recombinant virus carries 1.3 copies of the HBV genome (genotype D, serotype ayw) and is packaged in AAV serotype 8 capsids. Generation of the AAV-HBV carrier mouse model used in this study has been described previously (6, 24). Briefly, mice were injected with the AAV-HBV1.3 virus through the tail vein (1 × 10^11^ viral genome copies per mouse). After 5 weeks or more, stable HBV carrier mice were used.

### Statistics.

All statistical analysis was performed using GraphPad Prism software. For comparing 2 groups, *P* values were determined using an unpaired 2-tailed Student’s *t* test. For comparing more than 2 groups, 1-way ANOVA followed by Tukey’s post hoc test was applied. Data are shown as the mean ± SEM or mean + SEM. *P* < 0.05 was considered statistically significant.

### Study approval.

All animal experimental procedures were approved (no. ABSL-2-2022013) by the Biomedical Research Ethics Committee of the Institute of Biophysics, Chinese Academy of Sciences.

### Data availability.

All data relevant to this study are present in the article or the Supplemental Materials. Source data for this work are provided in the Supporting Data Values file.

## Author contributions

YXF, HP, and CYM conceived and designed the experiments; HP and YXF supervised the project; CYM, YL, and LX performed all the experiments; CYM, HP, and YXF analyzed the data; HL, JG, and HX performed or contributed to specific experiments; FW provided essential materials and helpful suggestions; CYM, HP, and YXF wrote the original draft of the manuscript; YXF, HP, CYM, YL, LX, and HL reviewed and edited the manuscript.

## Funding support

- National Natural Science Foundation of China (32300768 to CYM)
- Major Program of Guangzhou National Laboratory (GZNL2025C01036 to HP)
- National Key R&D Program of China (2018ZX10301-404 to HP)

## Supplementary Material

Supplemental data

Unedited blot and gel images

Supporting data values

## Version 1 12/08/2025

Electronic publication
