# Supplementary material for: Anti–PD-L1–IFN-α–adjuvanted HBsAg vaccine overcomes HBV immune tolerance through targeting both DCs and macrophages
Source: JCI Insight. 2025 Dec 8;10(23):e198097. doi: 10.1172/jci.insight.198097 (PMC12890514; doi:10.1172/jci.insight.198097)
Supplement: Supplemental data [file jciinsight-10-198097-s179.pdf]

Anti-PD-L1-IFNα-adjuvanted HBsAg vaccine overcomes HBV immune tolerance through targeting both DCs and macrophages

Chao-Yang Meng, Yong Liang, Longxin Xu, Hongjia Li, Jingya Guo, Hairong Xu, Fan Wang, Yang-Xin Fu and Hua Peng

Supplementary Materials and Methods

Construction of bone marrow chimaera

WT C57BL/6J mice (8 weeks old) were irradiated with a 60Co Gammacell source at 10 Gy (1 Gy/min). 5×10^6 bone marrow cells from donor mice (CD11c-DTR mice) were intravenously transferred to each mouse 24 hours after irradiation. The chimaeras were given prophylactic water containing antibiotics for 4 weeks following irradiation. Then, 8-10 weeks after bone marrow transplantation, the CD11c-DTR bone marrow chimeric mice were either used for immunization to detect IgM and IgG or injected with the AAV-HBV1.3 virus through the tail vein (1×10^{11} viral genome copies per mouse). 5 weeks after virus infection, the stable HBV carrier CD11c-DTR bone marrow chimeric mice were selected for immunotherapy study.

DC and macrophage depletion

For DC depletion, HBV carrier CD11c-DTR or CD11c-DTR bone marrow chimeric mice were injected intraperitoneally with DT (diphtheria toxin; Millipore, Darmstadt, Germany) at a dose of 4 ng per gram of body weight, 24 hours before assessment of depletion efficiency or immunization. To maintain the depletion, DT was administered every two days.

For macrophage depletion in inguinal LNs, HBV carrier or WT mice were injected subcutaneously with 40 μL of clodronate liposomes (CLL; FormuMax)

per mouse, starting 7-10 days before immunization. To maintain the depletion, CLL was administered every three days.

Cloning, expression, and purification of fusion proteins

Generation of aPD-L1-IFNα fusion protein has been previously described (1, 2), with site-directed mutations introduced in this study to prevent FcγR-mediated effector function. Briefly, mutations based on Eu nomenclature L234A, L235A, and P329G ('LALA-PG' mutants) were introduced into the CH2 domain of each of the IgG1 heavy chains (3-5). Then, the fusion protein with no FcγR binding was generated by transient co-transfection of two arms of plasmids into FreeStyle™ 293-F cells. The supernatant was collected on day 7 post-transfection, and the fusion protein was purified using a Protein A-Sepharose column according to the manufacturer's protocol (Repligen Corporation, USA). The heterogeneity and purity were confirmed by HPLC and SDS-PAGE. Both anti-PD-L1 (aPD-L1) antibody and IFNα-Fc fusion protein, which lack FcγR binding, were generated and purified using the same protocol. The concentrations of the purified proteins were determined by measuring the optical density at 280 nm.

In vitro activation assay of DC2.4 cells

DC2.4, an immortalized dendritic cell line derived from C57BL/6 mice, was kindly provided by Dr. Li Tang (Key Laboratory of Protein Engineering, Academy

of Military Medical Sciences, Beijing, China). To evaluate the effects of fusion proteins on dendritic cell activation *in vitro*, DC2.4 cells (1×10^5 cells/well) were seeded in 24-well plates. The cells were treated with either aPD-L1 (0.063 μg/mL), IFNα-Fc (0.039 μg/mL), a combination of aPD-L1 and IFNα-Fc, or the aPD-L1-IFNα fusion protein (0.1 μg/mL) with concentrations based on equimolar amount of aPD-L1 and IFNα subunits. The treatment was conducted at 37°C for 12 hours. Then, the expression levels of MHC I, CD80, and CD86 on DC2.4 cells were detected and analyzed using an LSRFortessa flow cytometer (BD Biosciences, USA) and FlowJo software (TreeStar), respectively.

Immunization

In this study, a low dose of aPD-L1-IFNα fusion protein was utilized as an immunological adjuvant, mixed with rHBsAg, and administered via subcutaneous immunization to HBV carrier mice using a prime-boost vaccination strategy. Briefly, aPD-L1-IFNα (2 μg/mouse) or other fusion proteins (aPD-L1 and IFNα-Fc, with equimolar amounts of aPD-L1 and IFNα as determined by the single subunit) were mixed with rHBsAg (1 μg/mouse) and injected subcutaneously into HBV carrier mice. Mice were administered the same dose of the mixture 7 days after the initial immunization, followed by booster immunizations with rHBsAg (1 μg/mouse) alone at 3 and 5 weeks post-initial immunization. The rHBsAg (1 μg/mouse) alone was injected subcutaneously into HBV carrier mice on days 0, 7, 21 and 35 as the control

vaccine group (rHBsAg). The rHBsAg vaccine was purchased from Dalian Hissen Bio-pharm Inc. (Dalian, China). Blood was collected from the retro-orbital sinuses at the indicated time points in the indicated experiments to monitor the levels of HBsAg, ayw subtype-specific anti-HBsAg IgG antibodies, and HBV-DNA in the serum.

Enzyme-linked immunosorbent assay (ELISA)

ELISA kits for HBsAg testing were purchased from Shanghai Kehua Bio-engineering Co., Ltd. (Shanghai, China). Serum levels of HBsAg were measured according to the manufacturer's instructions. The peptides for ELISA to test ayw subtype-specific anti-HBsAg IgG or IgM in sera, corresponding to amino acids 111-140 (PGSSTTSTGPCRTCMTTAQGTSMYPSCCCT, subtype ayw) with the same encoding sequence in AAV-HBV1.3, were synthesized by China Peptides Co., Ltd. (Shanghai, China). 96-well plates (Corning 9018, USA) were coated with the 111-140 amino acid peptides at 5 μg/mL (50 μL/well) in PBS and incubated at 4°C overnight. After blocking with blocking buffer (PBS containing 5% fetal bovine serum, FBS), serum samples were added at 1:100 dilution for IgG detection or 1:10 dilution for IgM detection. Then, horseradish peroxidase (HRP)-conjugated goat anti-mouse IgG (CWbio, Beijing, China) or HRP-conjugated goat anti-mouse IgM (Elabscience, Wuhan, China) was used for chromogenic reaction. The absorbance at 450-630 nm was detected using a microplate reader (Molecular Devices, USA).

HBV DNA and RNA detection

Serum HBV-DNA, liver HBV DNA and liver HBV RNA levels were measured by real-time PCR, as previously described (2, 6, 7).

Enzyme-linked immunospot assay (ELISpot)

Splenocytes were collected, and the erythrocytes were lysed with ammonium-chloride-potassium (ACK) buffer. The suspension was washed with RPMI 1640 medium and passed through a 70-μm cell strainer (Biologix Group) to obtain a single-cell suspension. For B cell ELISpot assay, 10 μg/mL HBsAg (subtype ayw, 50 μL/well) was coated onto 96-well ELISpot plates (BD Biosciences, USA) and incubated at 4 °C overnight. After blocking with RPMI 1640 medium containing 10% FBS, 1×10^6 lymphocytes from spleen in 100 μL of complete RPMI 1640 medium were added to the wells. After 6 hours incubation at 37 °C, HBsAg-specific IgG was analyzed using biotinylated donkey anti-mouse IgG (CWbio, Beijing, China) and streptavidin-HRP (BD Biosciences, USA). Antigen-specific T cell responses were tested by a T cell ELISpot assay. 1×10^6 lymphocytes from spleen were incubated for 48 hours at 37 °C in complete RPMI 1640 medium containing 5 μg/mL HBsAg (subtype ayw) or 10 μg/mL ENV190-197 peptides (VWLSVIWM; an HBsAg-specific CD8 peptide) in an IFN-γ ELISpot plate (BD Biosciences, USA). After incubation, the IFN-γ secretion was analyzed using biotinylated anti-mouse IFN-γ antibody and

streptavidin-HRP (BD Biosciences, USA). The spots were visualized with 3-amino-9-ethylcarbazole (AEC) substrate (BD Biosciences, USA) and were quantified with the ImmunoSpot Analyzer (Cellular Technology Ltd., USA). A recombinant HBsAg (subtype ayw) protein with the same encoding sequence as that in AAV-HBV1.3 was purchased from Meridian Life Science Inc. (Memphis, USA).

Flow cytometry analysis

LNs were harvested, gently minced using scissors in RPMI 1640 medium, and then digested with collagenase IV (1 mg/mL; Roche) and DNase I (100 μg/mL; Roche) at 37 °C for 30 min, followed by passing through a 70-μm cell strainer (Biologix Group) to generate a single-cell suspension.

For aPD-L1-IFNα binding analysis, the single cells from HBV carrier mice, *Pdl1*$^{-/-}$ HBV carrier mice, or DC *Pdl1*$^{-/-}$ HBV carrier mice were suspended in FACS buffer (PBS containing 2% FBS), blocked with anti-CD16/32 (clone 2.4G2, produced in-house) to prevent non-specific binding, and incubated with aPD-L1-IFNα (human IgG1 Fc) at the indicated concentrations, followed by incubation with fluorescently labeled antibodies and anti-human IgG Fc PE-Cy7 (M1310G05, BioLegend). For analysis of HBsAg-FITC uptake and costimulatory molecule expression, single cells were suspended in FACS buffer, blocked with anti-CD16/32 (clone 2.4G2, produced in-house), and incubated with fluorescently labeled antibodies. The antibodies used included anti-CD3ℇ

APC (145-2C11, eBioscience), anti-CD4 FITC (GK1.5, eBioscience), anti-CD4 PerCP-Cy5.5 (RM4-5, eBioscience), anti-CD8α PE (53-6.7, BioLegend), anti-CD8α PE-Cy7 (53-6.7, eBioscience), anti-B220 BV650 (RA3-6B2, BioLegend), anti-CD11c APC-Cy7 (HL3, BD Pharmingen™), anti-CD11c APC (N418, Invitrogen), anti-MHC II Alexa Fluor® 700 (M5/114.15.2, eBioscience), anti-MHC I PerCP-eFluor® 710 (SF1-1.1.1, eBioscience), anti-CD11b PerCP-Cy5.5 (M1/70, eBioscience), anti-CD103 BV711 (2E7, Biolegend), anti-F4/80 PE (BM8, eBioscience), anti-F4/80 PE-Cy7 (BM8, Invitrogen), anti-CD169 APC (3D6.112, Biolegend), anti-CD80 FITC (16-10A1, Tonbo), anti-CD86 PE (GL1, eBioscience), anti-PD1 APC (J43, eBioscience), anti-CXCR5 PE (SPRCL5, eBioscience), anti-GL7 AF488 (GL-7, eBioscience), anti-FAS PE-Cy7 (Jo2, BD Pharmingen™), anti-PD-L1 PE-Cy7 (10F.9G2, BioLegend). DAPI (Invitrogen) was used to exclude dead cells. Data were acquired and analyzed using an LSRFortessa flow cytometer (BD Biosciences, USA) and FlowJo software (TreeStar), respectively.

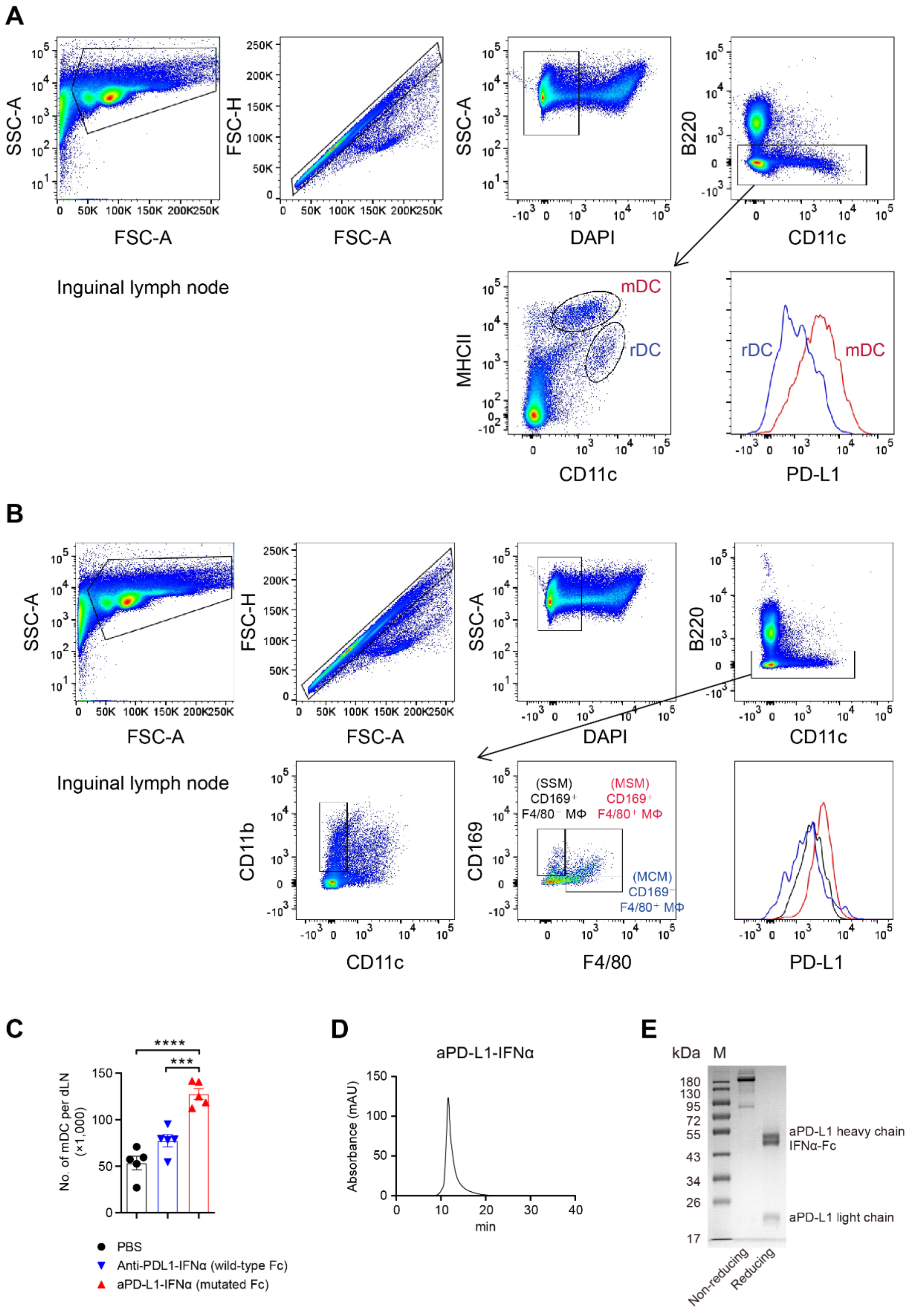

Supplemental Figure 1. Gating strategy for PD-L1 expression on DCs and macrophages and aPD-L1-IFNα characterization. (**A**) Gating strategy for the expression of PD-L1 on mDCs and rDCs in inguinal LN from HBV carrier mouse.

(**B**) Gating strategy for the expression of PD-L1 on SSMs, MSMs and MCMs in inguinal LN from HBV carrier mouse. (**C**) A single dose subcutaneous injection of anti-PDL1-IFNα (wild-type Fc, 2 μg/mouse), aPD-L1-IFNα (mutated Fc, 2 μg/mouse) or PBS into HBV carrier mice (n = 5/group). 3 days later, mDCs in inguinal dLNs were analyzed by flow cytometry. (**D** and **E**) The aPD-L1-IFNα (mutated Fc) heterodimeric fusion protein was expressed in 293F cells, and the purified protein was subsequently analyzed by HPLC (**D**) and SDS-PAGE (**E**), respectively. Data are shown as the mean±SEM (**C**) and are representative of at least two independent experiments (**C**-**E**). One-way analysis of variance (ANOVA) followed by Tukey's test was applied in (**C**). ****P* < 0.001; *****P* < 0.0001. Fc, fragment crystallizable; MΦ, macrophage; M, molecular weight marker; min, minutes.

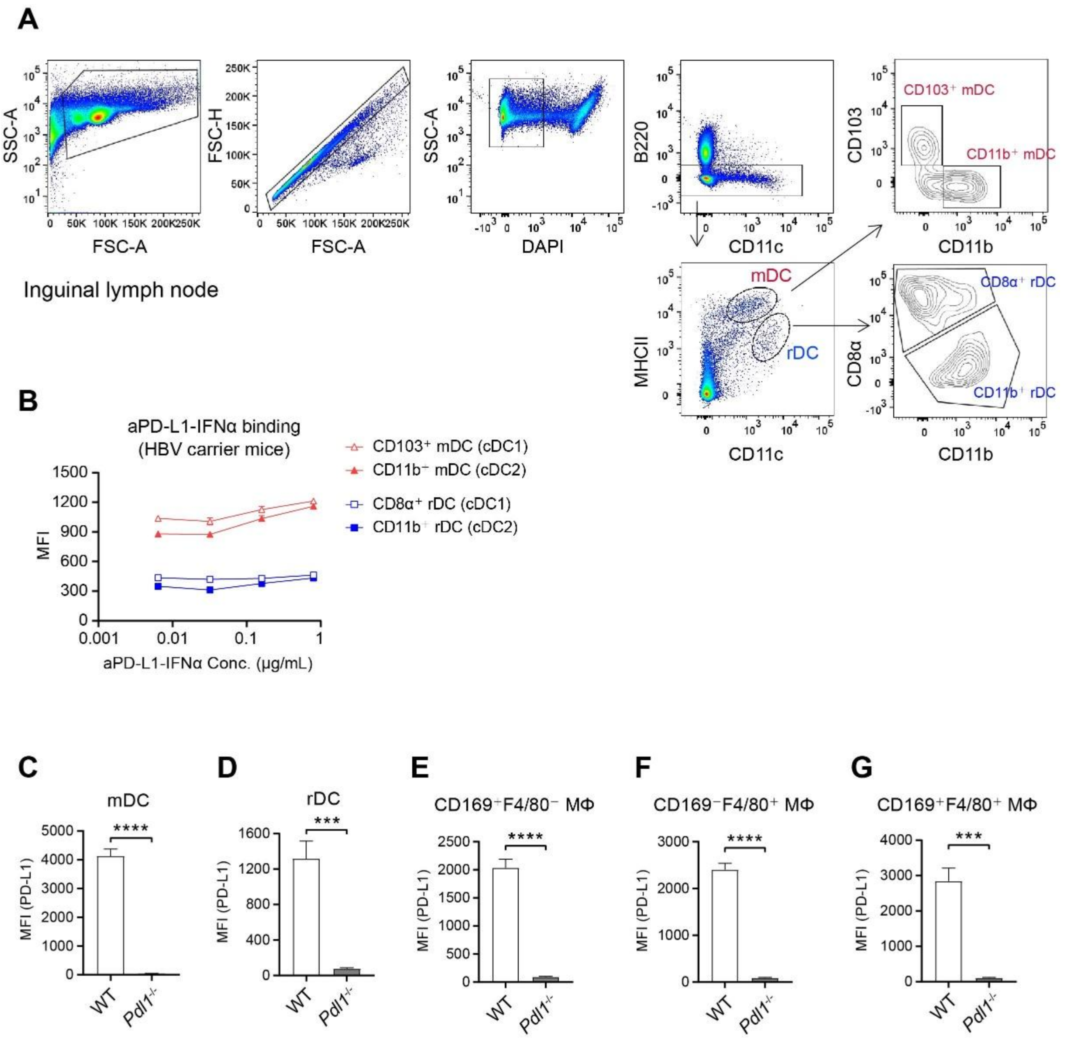

Supplemental Figure 2. aPD-L1-IFNα binding and PD-L1 knockout in *Pdl1*$^{-/-}$ mice. (**A**) Gating strategy for CD103^{+} mDCs, CD11b^{+} mDCs, CD8α^{+} rDCs and CD11b^{+} rDCs in inguinal LN from HBV carrier mouse. (**B**) The single cells from inguinal LNs of HBV carrier mice (*n* = 4) were collected and incubated with aPD-L1-IFNα (human IgG1 Fc) at the indicated concentrations, followed by incubation with fluorescently labeled antibodies and anti-human IgG Fc (PE-Cy7 label) *in vitro*. The MFI of PE-Cy7 was detected by flow cytometry. (**C**-**G**) The single cells from inguinal LNs of WT mice or *Pdl1*$^{-/-}$ mice (*n* = 4/group) were collected and the expression of PD-L1 was analyzed by flow cytometry. PD-L1

expression levels on mDCs (**C**), rDCs (**D**), $CD169^+F4/80^-$ MΦ (**E**), $CD169^-F4/80^+$ MΦ (**F**) and $CD169^+F4/80^+$ MΦ (**G**) are shown. Data are shown as the mean±SEM (**B**) or mean+SEM (**C**-**G**) and are representative of at least two independent experiments. An unpaired two-tailed Student's *t*-test was applied in (**C**-**G**). ***$P < 0.001$; ****$P < 0.0001$. Conc., concentrations; MΦ, macrophage; MFI, mean fluorescence intensity.

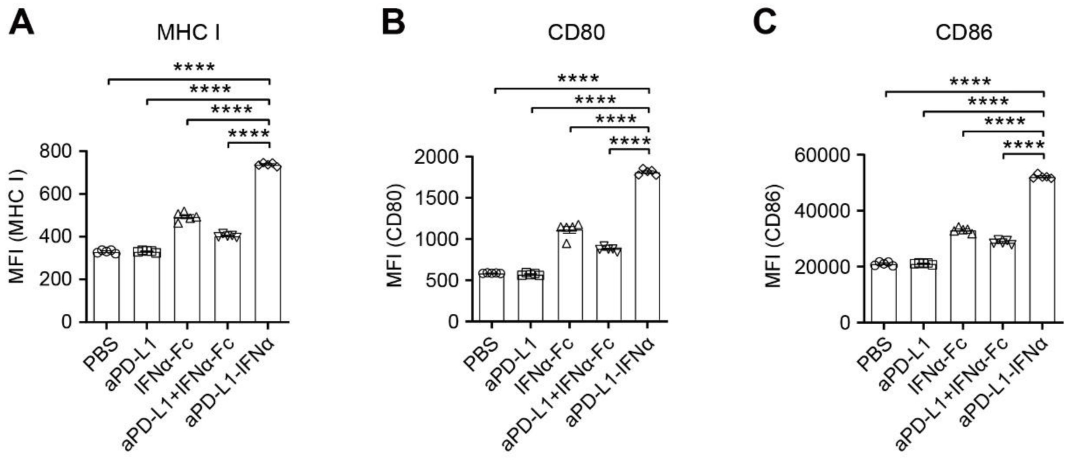

Supplemental Figure 3. aPD-L1 *cis*-binding of low-affinity IFNα activates DC2.4 cells. (**A**-**C**) DC2.4 cells (1×10^5 cells/well) were incubated with equimolar amounts of aPD-L1, IFNα-Fc, a combination of aPD-L1 and IFNα-Fc, or aPD-L1-IFNα for 12 hours. The expression levels of MHC I (**A**), CD80 (**B**), and CD86 (**C**) on DC2.4 cells were detected by flow cytometry ($n = 5$ culture wells). Data are shown as the mean±SEM and are representative of at least two independent experiments. One-way ANOVA followed by Tukey's test was applied in (**A**-**C**). ****$P < 0.0001$. MFI, mean fluorescence intensity.

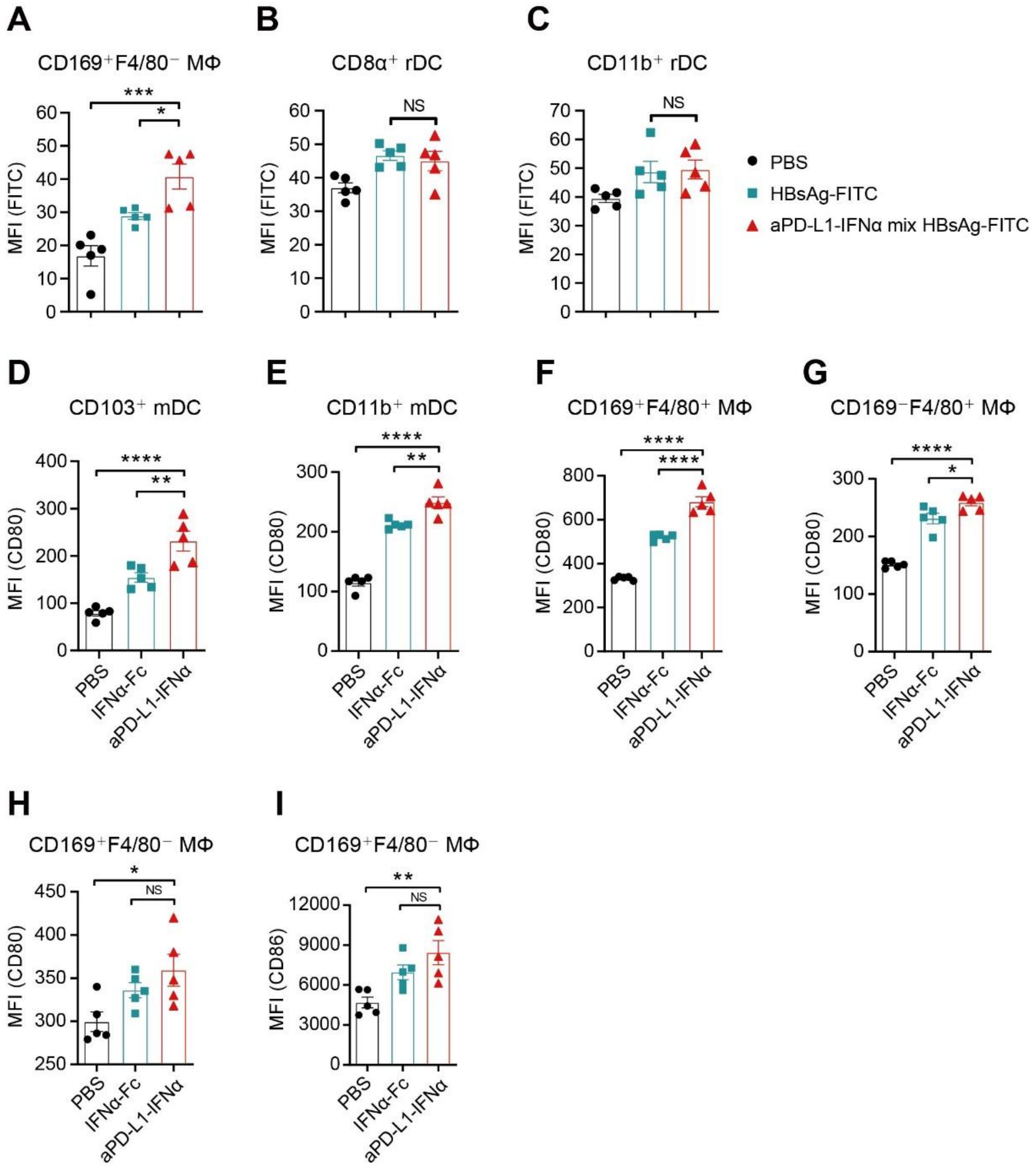

Supplemental Figure 4. Effect of aPD-L1-IFNα on antigen uptake and activation of APCs. (**A**-**C**) subcutaneous injection of HBsAg-FITC (1 μg/mouse) mix or without aPD-L1-IFNα (2 μg/mouse) into HBV carrier mice (*n* = 5/group). 6 hours post-injection, the uptake of HBsAg-FITC by macrophages and rDCs in inguinal dLNs was analyzed using flow cytometry. The uptake of HBsAg-FTIC by CD169$^+$F4/80$^-$ MΦ (**A**), CD8α$^+$ rDCs (**B**) and CD11b$^+$ rDCs (**C**) is shown. (**D**-**I**) A single dose subcutaneous injection of IFNα-Fc (0.78 μg/mouse) or aPD-L1-

IFNα (2 μg/mouse) (determined by equimolar of IFNα subunit) into HBV carrier mice (*n* = 5/group). After 24 hours, the expression of CD80 and CD86 on DCs and macrophages in inguinal dLNs was analyzed by flow cytometry. The expression of CD80 on $CD103^+$ mDCs (**D**), $CD11b^+$ mDCs (**E**), $CD169^+F4/80^+$ MΦ (**F**), $CD169^-F4/80^+$ MΦ (**G**) and $CD169^+F4/80^-$ MΦ (**H**) is shown. The expression of CD86 on $CD169^+F4/80^-$ MΦ (**I**) is shown. Data are shown as the mean±SEM and are representative of at least two independent experiments. One-way ANOVA followed by Tukey's test was applied in (**A**-**I**). *$P < 0.05$; **$P < 0.01$; ***$P < 0.001$; ****$P < 0.0001$. FITC, fluorescein isothiocyanate; MΦ, macrophage; MFI, mean fluorescence intensity; NS, not significant.

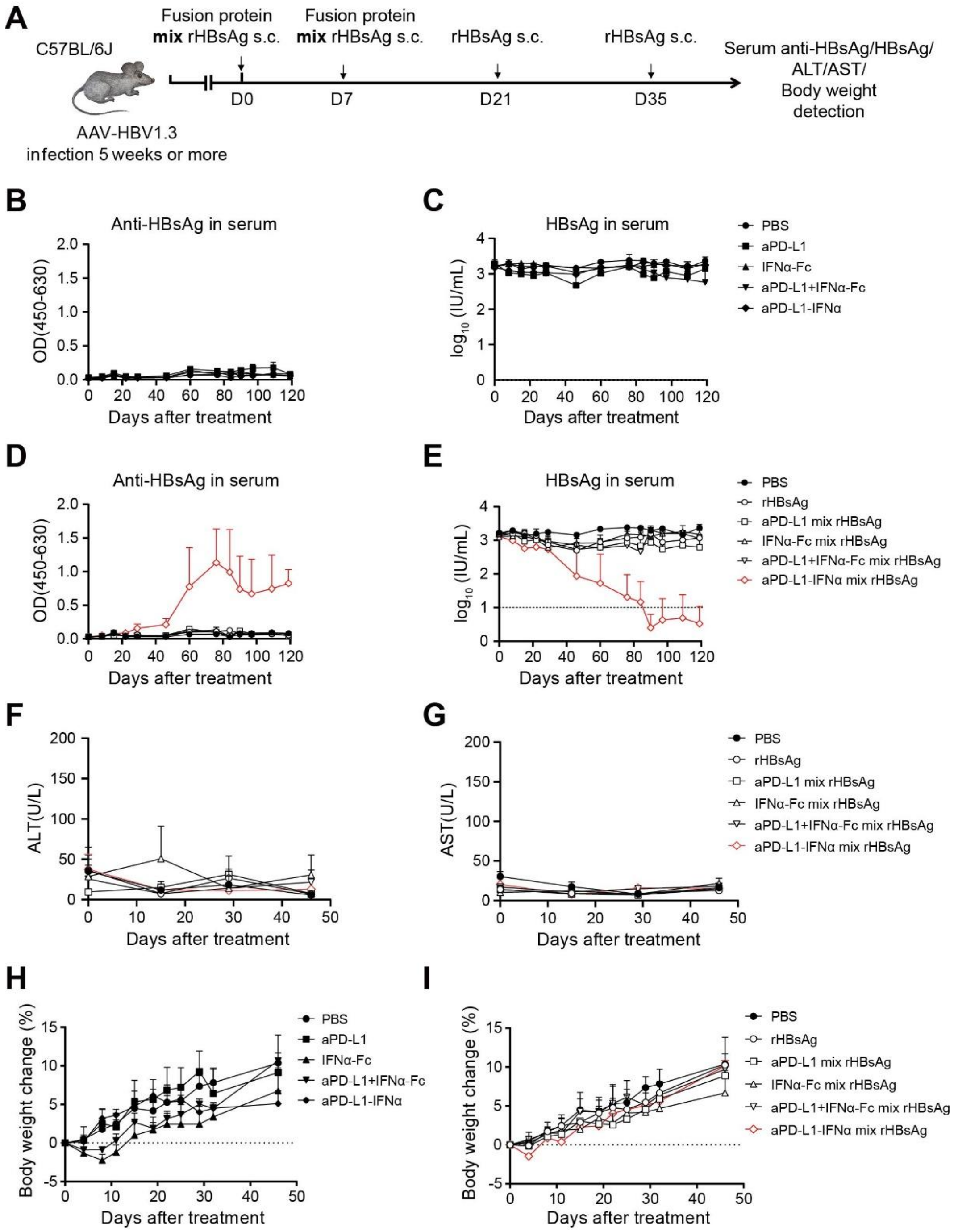

Supplemental Figure 5. aPD-L1-IFNα, as a targeted adjuvant, exerts superior anti-HBV effect. (**A**) Time schedule for the fusion protein adjuvant mixed with rHBsAg as a therapeutic vaccine (co-immunization) to treat HBV carrier mice. aPD-L1 (1.22 μg/mouse), IFNα-Fc (0.78 μg/mouse), aPD-L1 plus

IFNα-Fc, or aPD-L1-IFNα (2 μg/mouse) (determined by equimolar of aPD-L1 and IFNα subunits) was mixed with rHBsAg (1 μg/mouse) and injected subcutaneously into HBV carrier mice. A second immunization with the same mixture was given 7 days after the initial immunization, followed by booster immunizations with rHBsAg (1 μg/mouse) alone at 3 and 5 weeks post-initial immunization. The rHBsAg group received rHBsAg (1 μg/mouse) subcutaneous injection into HBV carrier mice on days 0, 7, 21 and 35. aPD-L1, IFNα-Fc, aPD-L1 plus IFNα-Fc, or aPD-L1-IFNα alone was administered subcutaneously into HBV carrier mice on days 0 and 7 as the adjuvant-alone group. (**B** and **C**) The sera from HBV carrier mice treated with fusion protein alone (*n* = 3/group) were collected. Serum levels of ayw subtype-specific anti-HBsAg IgG (**B**) and HBsAg (**C**) were examined by ELISA. (**D** and **E**) The sera from HBV carrier mice treated with the co-immunization (*n* = 3/group) were collected. Serum levels of ayw subtype-specific anti-HBsAg IgG (**D**) and HBsAg (**E**) were examined by ELISA. The detection limit in panel **E** is indicated by a dashed line. (**F** and **G**) Levels of ALT (**F**) and AST (**G**) in the serum (*n* = 3/group) were measured following the manufacturer's instructions (Nanjing Jiancheng Bioengineering Institute, Nanjing, China). (**H** and **I**) Body weight changes were monitored in HBV carrier mice (*n* = 3/group) treated with the fusion protein adjuvant alone (**H**) or co-immunization (**I**). Data are shown as the mean+SEM and are representative of at least two independent experiments. OD, optical density; s.c., subcutaneous.

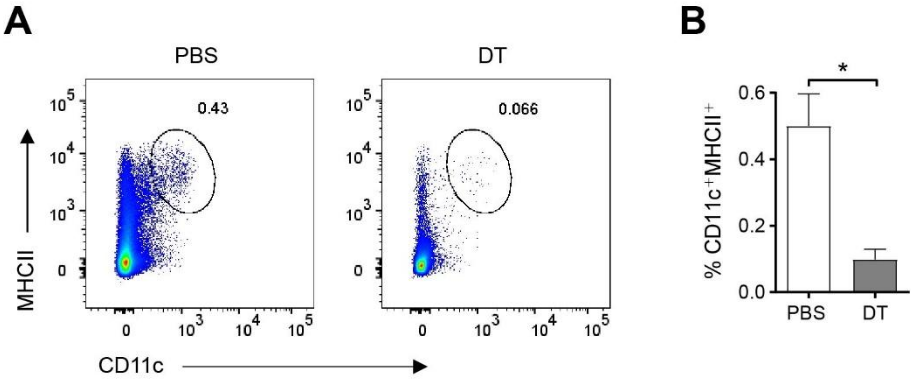

Supplemental Figure 6. *In vivo* DCs depletion. (**A** and **B**) The percentages of DCs in the spleen of HBV carrier CD11c-DTR bone marrow chimeric mice were detected by flow cytometry 24 hours after DT (4 ng/g body weight) or PBS intraperitoneal injection (n = 3/group). Representative flow cytometry plots (gated cells from single cells, live, $B220^-$) (**A**) and percentages (**B**) of DCs ($CD11c^+MHCII^+$) in the spleens are shown. Data are shown as the mean+SEM and are representative of at least two independent experiments. An unpaired two-tailed Student's *t*-test was applied. *$P < 0.05$.

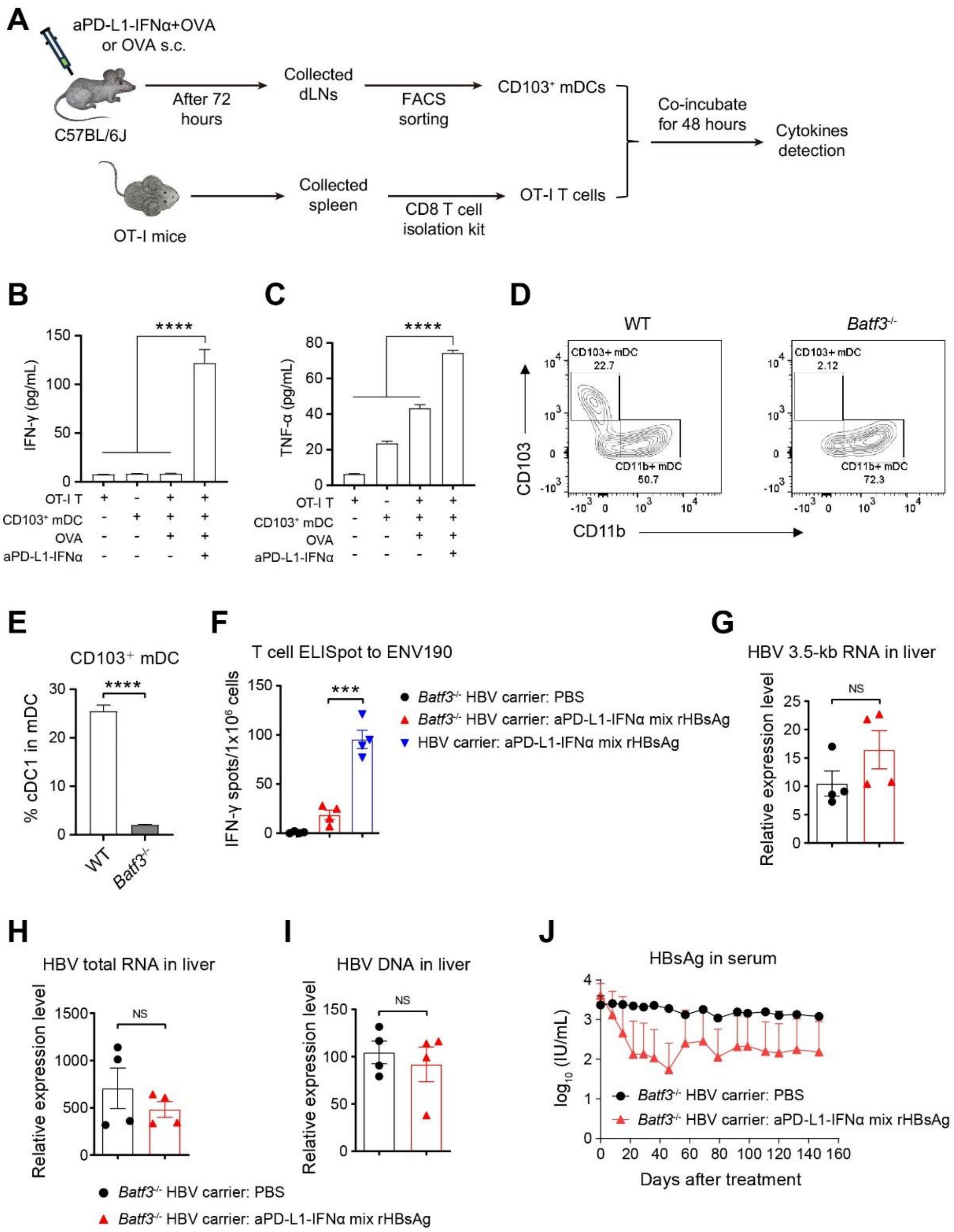

Supplemental Figure 7. aPD-L1-IFNα significantly promotes CD103⁺ mDCs cross-presentation. (**A**) Schematic diagram for detecting the antigen cross-presentation function of CD103⁺ mDCs. WT mice were subcutaneously immunized with either a mixture of aPD-L1-IFNα (2 μg/mouse) and ovalbumin (OVA) (1 μg/mouse) or OVA (1 μg/mouse) alone. 72 hours after immunization,

CD103$^+$ mDCs were sorted from inguinal dLNs by flow cytometry. OT-I T cells were sorted from OT-I TCR transgenic mice by mojosort^{TM} mouse CD8 T cell isolation kit (Biolegend). Then, CD103$^+$ mDCs (1×10^4 cells/well) incubated with OT-I T cells (1×10^5 cells/well) for 48 hours. The supernatants were harvested, and levels of IFN-γ and TNF-α were measured using cytometric bead array (CBA). (**B** and **C**) The supernatants from OT-I T cells alone, CD103$^+$ mDCs alone, OT-I T cells incubated with CD103$^+$ mDCs (sorted from OVA alone immunized mice), and OT-I T cells incubated with CD103$^+$ mDCs (sorted from aPD-L1-IFNα plus OVA co-immunization mice) were harvested (*n* = 4-5 culture wells), and levels of IFN-γ (**B**) and TNF-α (**C**) were measured using CBA. (**D** and **E**) The percentages of CD103$^+$ mDCs in inguinal LNs of WT mice or *Batf3*$^{-/-}$ mice (*n* = 4/group) were detected by flow cytometry. Representative flow cytometry plots (gated cells from single cells, live, B220$^-$, CD11c$^+$MHCII^{hi}) (**D**) and percentages (**E**) of CD103$^+$ mDCs are shown. (**F**) *Batf3*$^{-/-}$ HBV carrier mice or HBV carrier mice were treated as described in Figure 3A. A total of 1×10^6 splenocytes from each mouse (*n* = 4/group) were collected. ENV190-specific CD8$^+$ T cell responses were tested by a T cell ELISpot assay. (**G**-**J**) *Batf3*$^{-/-}$ HBV carrier mice were treated as described in Figure 3A. Levels of HBV intermediate products, including HBV 3.5-kb RNA (**G**), HBV total RNA (**H**), and HBV DNA (**I**) in the liver, were measured by real-time PCR on day 147 (*n* = 4/group). Serum levels of HBsAg (*n* = 4/group) were examined by ELISA (**J**). Data are shown as the mean+SEM (**B**, **C**, **E**, **J**) or mean±SEM (**F**-**I**) and are representative of at

least two independent experiments. One-way ANOVA followed by Tukey's test was applied in (**B**, **C**). An unpaired two-tailed Student's *t*-test was applied in (**E**-**I**). ***P < 0.001; ****P < 0.0001. NS, not significant; s.c., subcutaneous.

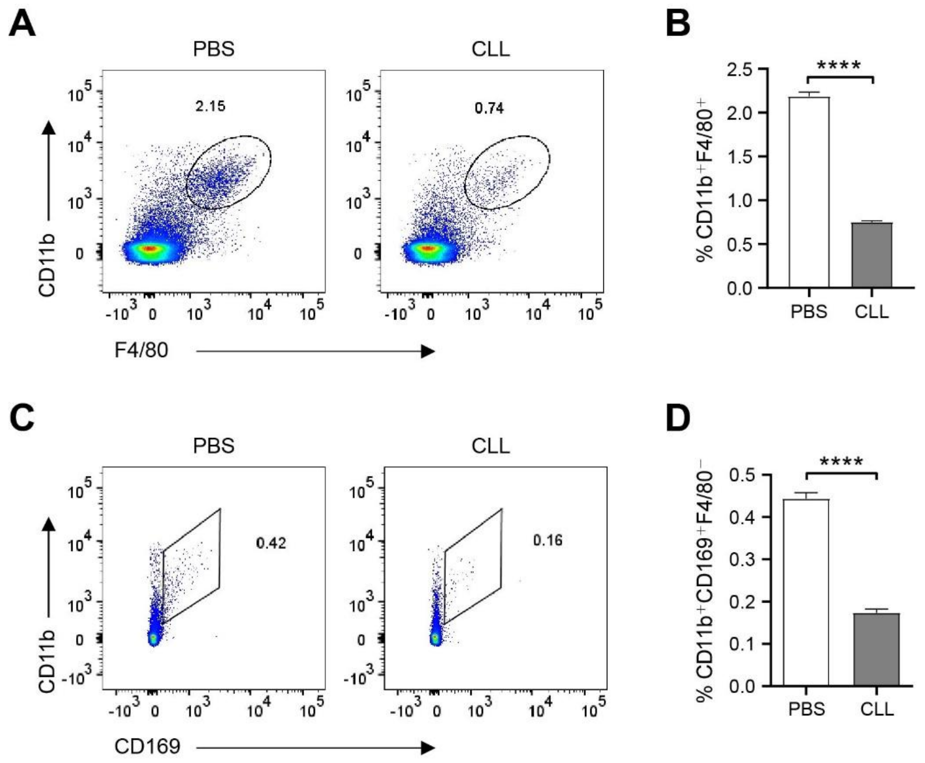

Supplemental Figure 8. *In vivo* mcrophages depletion. (**A** and **B**) The percentages of CD11b$^+$F4/80$^+$ MΦ in inguinal dLNs of HBV carrier mice were detected by flow cytometry 48 hours after CLL (40 μL/mouse) or PBS subcutaneous injection (n = 3/group). Representative flow cytometry plots (gated cells from single cells, live, B220$^-$, CD11c$^-$) (**A**) and percentages (**B**) of CD11b$^+$F4/80$^+$ MΦ are shown. (**C** and **D**) The percentages of CD11b$^+$CD169$^+$F4/80$^-$ MΦ in inguinal dLNs of HBV carrier mice were detected by flow cytometry 48 hours after CLL (40 μL/mouse) or PBS subcutaneous injection (n = 3/group). Representative flow cytometry plots (gated cells from single cells, live, B220$^-$, CD11c$^-$, F4/80$^-$) (**C**) and percentages (**D**) of CD11b$^+$CD169$^+$F4/80$^-$ MΦ are shown. Data are shown as the mean+SEM (**B**, **D**) and are representative of at least two independent experiments. An unpaired

two-tailed Student's *t*-test was applied in (**B**, **D**). ****$P < 0.0001$.

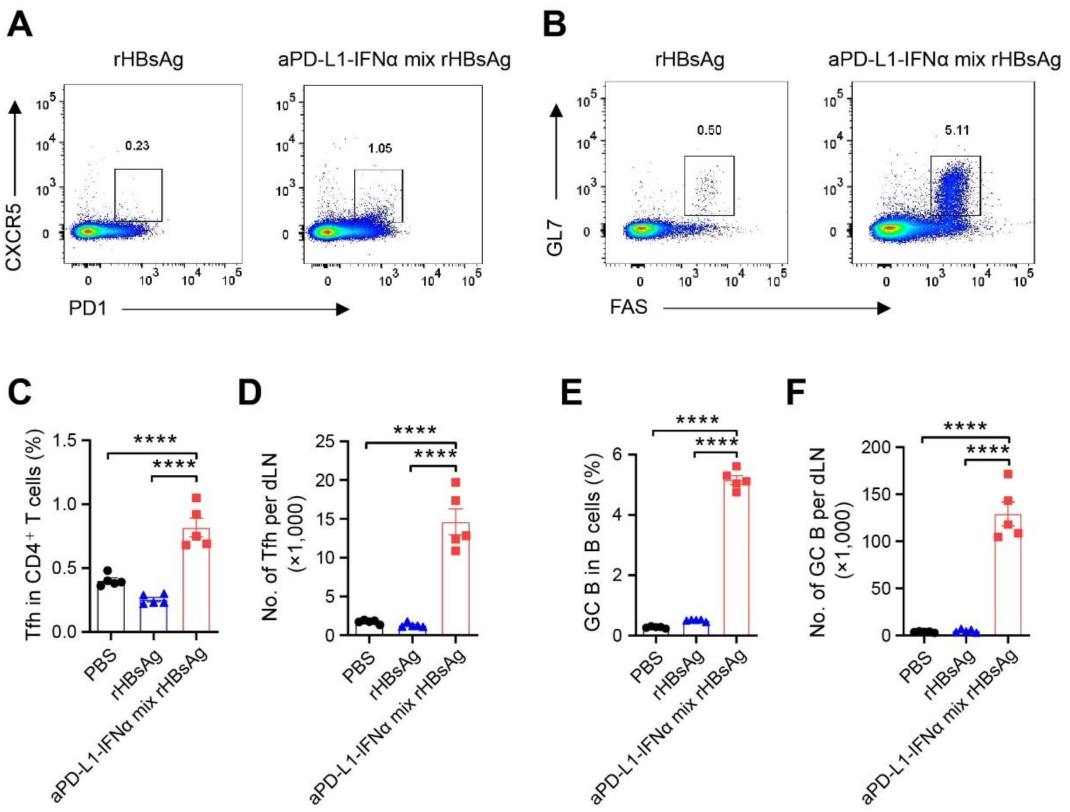

Supplemental Figure 9. The co-immunization efficiently stimulates Tfh and GC B generation. (**A**, **C**, **D**) HBV carrier mice (*n* = 5/group) were injected subcutaneously with PBS, rHBsAg (1 μg/mouse), or aPD-L1-IFNα (2 μg/mouse) plus rHBsAg (1 μg/mouse), and the inguinal dLNs were harvested 5 days after the initial immunization. Tfh cells from dLNs were analyzed by flow cytometry. Representative flow cytometry plots (gated cells from single cells, live, $CD4^+$) (**A**), percentages (**C**) and quantification (**D**) of Tfh cells ($CD4^+CXCR5^+PD1^+$) are shown. (**B**, **E**, **F**) HBV carrier mice (*n* = 5/group) were injected subcutaneously with PBS, rHBsAg (1 μg/mouse), or aPD-L1-IFNα (2 μg/mouse) plus rHBsAg (1 μg/mouse) on days 0 and 7, and the inguinal dLNs were harvested 10 days after the second immunization. GC B cells from dLNs were analyzed by flow

cytometry. Representative flow cytometry plots (gated cells from single cells, live, B220⁺) (**B**), percentages (**E**) and quantification (**F**) of GC B cells ($B220^+FAS^+GL7^+$) are shown. Data are shown as the mean±SEM and are representative of at least two independent experiments. One-way ANOVA followed by Tukey's test was applied in (**C**-**F**). ****P < 0.0001.

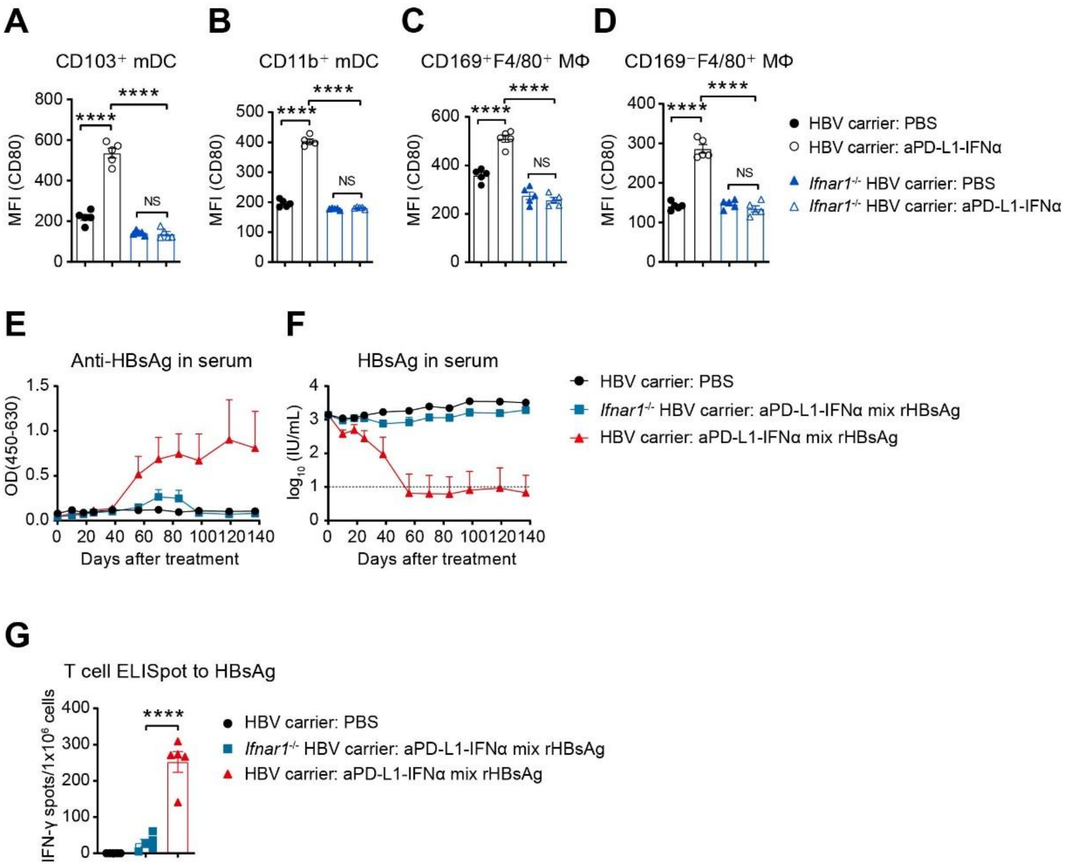

Supplemental Figure 10. aPD-L1-IFNα activates APCs through IFNAR signaling pathway. (**A**-**D**) HBV carrier mice or *Ifnar1*$^{-/-}$ HBV carrier mice (*n* = 5/group) were injected subcutaneously with a single dose of aPD-L1-IFNα (2 μg/mouse) or PBS. After 24 hours, the expression of CD80 on DCs and macrophages in inguinal dLNs was analyzed by flow cytometry. The expression of CD80 on CD103^{+} mDCs (**A**), CD11b^{+} mDCs (**B**), CD169^{+}F4/80^{+} MΦ (**C**) and CD169^{-}F4/80^{+} MΦ (**D**) is shown. (**E**-**G**) HBV carrier mice or *Ifnar1*$^{-/-}$ HBV carrier mice (*n* = 5/group) were treated as described in Figure 3A. Serum levels of ayw subtype-specific anti-HBsAg IgG (**E**) and HBsAg (**F**) were determined by ELISA. The detection limit in panel **F** is indicated by a dashed line. A total of 1×10^{6} splenocytes from each mouse were collected on day 137. Specific T cell

responses to HBsAg (subtype ayw) were tested by a T cell ELISpot assay (**G**). Data are shown as the mean±SEM (**A**-**D**, **G**) or mean+SEM (**E**, **F**) and are representative of at least two independent experiments. One-way ANOVA followed by Tukey's test was applied in (**A**-**D**). An unpaired two-tailed Student's *t*-test was applied in (**G**). ****$P < 0.0001$. MΦ, macrophage; MFI, mean fluorescence intensity; NS, not significant; OD, optical density.

References

1. Liang Y, et al. Targeting IFNα to tumor by anti-PD-L1 creates feedforward antitumor responses to overcome checkpoint blockade resistance. *Nat Commun.* 2018;9(1):4586.
2. Meng C-Y, et al. Engineered anti-PDL1 with IFNα targets both immunoinhibitory and activating signals in the liver to break HBV immune tolerance. *Gut.* 2023;72(8):1544-1554.
3. Schlothauer T, et al. Novel human IgG1 and IgG4 Fc-engineered antibodies with completely abolished immune effector functions. *Protein Eng Des Sel.* 2016;29(10):457-466.
4. Liu L, et al. Rejuvenation of tumour-specific T cells through bispecific antibodies targeting PD-L1 on dendritic cells. *Nat Biomed Eng.* 2021;5(11):1261-1273.
5. Andreata F, et al. CD8 cis-targeted IL-2 drives potent antiviral activity

against hepatitis B virus. *Sci Transl Med.* 2024;16(729):eadi1572.

6. Yan H, et al. Sodium taurocholate cotransporting polypeptide is a functional receptor for human hepatitis B and D virus. *eLife.* 2012;1:e00049.

7. Bian Y, et al. Vaccines targeting preS1 domain overcome immune tolerance in hepatitis B virus carrier mice. *Hepatology.* 2017;66(4):1067-1082.
