# Supplementary figures and images for: Anti–PD-L1–IFN-α–adjuvanted HBsAg vaccine overcomes HBV immune tolerance through targeting both DCs and macrophages
Source: JCI Insight. 2025 Dec 8;10(23):e198097. doi: 10.1172/jci.insight.198097 (PMC12890514; doi:10.1172/jci.insight.198097)

Full unedited gel for Supplemental Figure 1E

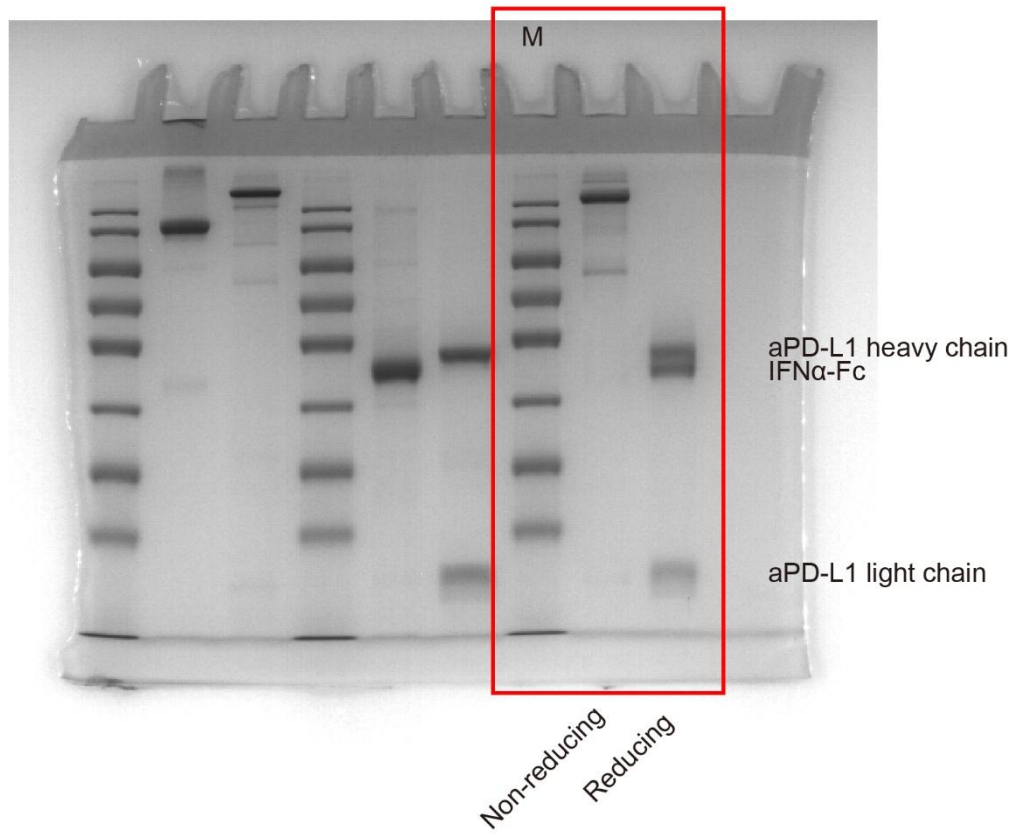

Supplement: Unedited blot and gel images [file jciinsight-10-198097-s180.pdf]
